# A Panel of Kaposi’s Sarcoma-Associated Herpesvirus Mutants in the Polycistronic Kaposin Locus for Precise Analysis of Individual Protein Products

**DOI:** 10.1128/jvi.01560-21

**Published:** 2022-03-09

**Authors:** Mariel Kleer, Grant MacNeil, Nancy Adam, Eric S. Pringle, Jennifer A. Corcoran

**Affiliations:** a Microbiology, Immunology, and Infectious Diseases Department, University of Calgary, Calgary, Alberta, Canada; b Charbonneau Cancer Institute, University of Calgary, Calgary, Alberta, Canada; c Snyder Institute for Chronic Diseases, University of Calgary, Calgary, Alberta, Canada; d Department of Microbiology and Immunology, Dalhousie University, Halifax, Nova Scotia, Canada; e Department of Biochemistry and Molecular Biology, University of Calgary, Calgary, Alberta, Canada; University of Arizona

**Keywords:** BAC16, KSHV, Kaposi’s sarcoma, Kaposi’s sarcoma-associated herpesvirus, kaposin B, kaposin locus, recombinant virus

## Abstract

Kaposi’s sarcoma-associated herpesvirus (KSHV) is the cause of several human cancers, including the endothelial cell (EC) malignancy, Kaposi’s sarcoma. Unique KSHV genes absent from other human herpesvirus genomes, the “K-genes,” are important for KSHV replication and pathogenesis. Among these, the kaposin transcript is highly expressed in all phases of infection, but its complex polycistronic nature has hindered functional analysis to date. At least three proteins are produced from the kaposin transcript: Kaposin A (KapA), B (KapB), and C (KapC). To determine the relative contributions of kaposin proteins during KSHV infection, we created a collection of mutant viruses unable to produce kaposin proteins individually or in combination. In previous work, we showed KapB alone recapitulated the elevated proinflammatory cytokine transcripts associated with KS via the disassembly of RNA granules called processing bodies (PBs). Using the new ΔKapB virus, we showed that KapB was necessary for this effect during latent KSHV infection. Moreover, we observed that despite the ability of all kaposin-deficient latent iSLK cell lines to produce virions, all displayed low viral episome copy number, a defect that became more pronounced after primary infection of naive ECs. For ΔKapB, provision of KapB in *trans* failed to complement the defect, suggesting a requirement for the *kaposin* locus in *cis*. These findings demonstrate that our panel of kaposin-deficient viruses enables precise analysis of the respective contributions of individual kaposin proteins to KSHV replication. Moreover, our mutagenesis approach serves as a guide for the functional analysis of other complex multicistronic viral loci.

**IMPORTANCE** Kaposi’s sarcoma-associated herpesvirus (KSHV) expresses high levels of the kaposin transcript during both latent and lytic phases of replication. Due to its repetitive, GC-rich nature and polycistronic coding capacity, until now no reagents existed to permit a methodical analysis of the role of individual kaposin proteins in KSHV replication. We report the creation of a panel of recombinant viruses and matched producer cell lines that delete kaposin proteins individually or in combination. We demonstrate the utility of this panel by confirming the requirement of one kaposin translation product to a key KSHV latency phenotype. This study describes a new panel of molecular tools for the KSHV field to enable precise analysis of the roles of individual kaposin proteins during KSHV infection.

## INTRODUCTION

Kaposi’s sarcoma-associated herpesvirus (KSHV) is the infectious cause of the endothelial cell (EC) neoplasm, Kaposi’s sarcoma (KS), and two rare lymphoproliferative disorders: primary effusion lymphoma (PEL) and multicentric Castleman disease (MCD) (1–3). Like all herpesviruses, KSHV establishes persistent, lifelong infection of its human host and displays two modes of infection: latent and lytic replication. Latency is the default replication program upon *de novo* infection in most cell types (4, 5). Following a transient period of lytic gene expression which serves to amplify genome copy number and evade the intrinsic immune response (6, 7), the viral episome is circularized and tethered to the host chromosome by the viral latency-associated nuclear antigen (LANA) (8). This results in formation of microscopically visible LANA nuclear bodies (NBs), which have been reported previously to correlate with intracellular viral genome copy number (9–13). In the latent state, the viral genome is passively replicated and unevenly partitioned to daughter cells by host cell machinery (10, 14, 15). The viral genome will remain in a highly chromatinized state until expression of the viral lytic switch protein, replication and transcription activator (RTA), which is both necessary and sufficient for lytic reactivation (16–18). Following reactivation, lytic gene expression follows a prescribed temporal cascade with genome replication marking the transition from early to late gene expression (19, 20). More recent analyses using single cell approaches show that lytic reactivation is quite heterogenous in terms of viral gene expression, host cell responses and outcomes (13, 21–23). The lytic replication phase produces progeny virions enabling transmission of the virus and culminates in cell death.

The KSHV-infected cells in KS lesions are predominantly proliferating endothelial cells (ECs) with an abnormal elongated or “spindled” morphology. The majority of these tumor ECs exhibit latent KSHV infection, whereas lytic replication is limited (24–27). Lytic replication is hypothesized to play a role in KS, likely due to the ongoing production of progeny virions as well as the release of inflammatory and angiogenic factors (14, 28, 29). Consistent with this, limiting viral lytic replication caused KS regression, suggesting that spontaneous lytic reactivation is required for ongoing infection of naive cells in the tumor environment and supports the cancer (30–32).

Since latently infected cells comprise the bulk of the KS lesion, the contribution of KSHV latent gene expression to tumorigenesis has been explored extensively in both animal and cell culture models (29, 33–37). *In vitro* infection of primary ECs with KSHV recapitulates many of the features of KS tumors, including efficient establishment of viral latency. During latency, gene expression is limited to six consensus protein products produced from an approximately 10kbp region of the viral genome termed the latency locus (LANA, viral cyclin [v-Cyclin], and viral fas-associated death domain [FADD]-like interleukin-1-β-converting enzyme [FLICE] inhibitory protein [v-FLIP], kaposins [Kap] A, B, and C) and 12 pre-miRNAs that are processed into at least 25 mature miRNAs (38–41). Using ectopic expression models, several latent gene products have been shown to contribute to the establishment and maintenance of viral latency as well as phenotypes associated with KS tumors, reviewed in references 42 and 43), but these studies have predominantly focused on LANA, v-Cyclin, and v-FLIP, while the contributions of the polycistronic kaposin locus are less clear. The kaposin mRNA was first identified as a marker of KSHV latent infection in KS tumors in 1997 (27), and it remains the most abundant viral transcript in KS tumor isolates (44). The kaposin locus comprises a significant fraction of KSHV coding capacity during latency and kaposin transcription is also upregulated during lytic replication, suggesting that this region of the viral genome is important (45). Despite this, we still know very little about the role of the kaposin locus, or the proteins it encodes, during viral replication.

**FIG 1:**
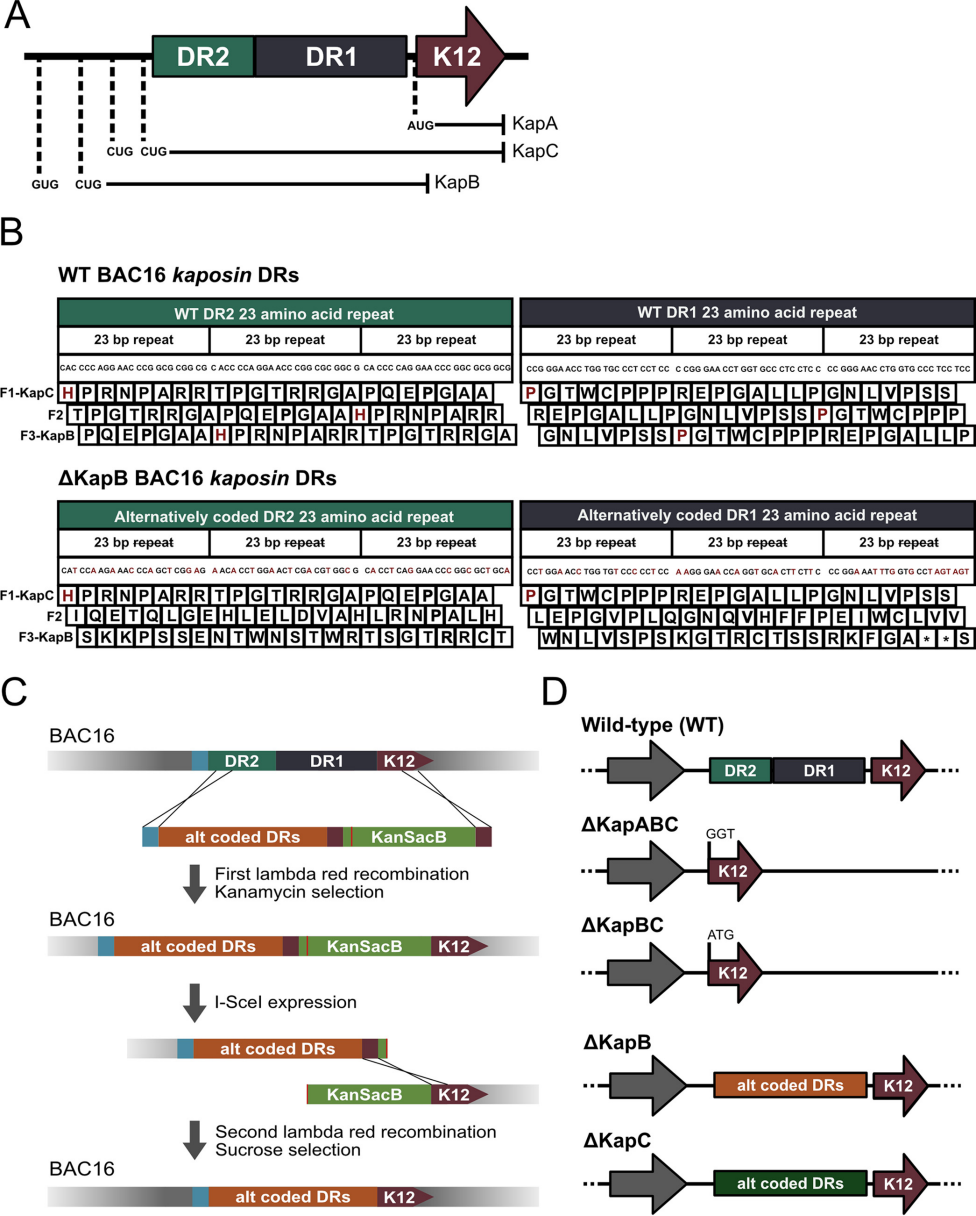
Lambda Red recombination facilitates the creation of four kaposin-deficient BAC16 DNA constructs. (A) A simplified version of the kaposin transcript is depicted in the 5′-to-3′ orientation. ORFs for kaposin A (KapA), kaposin B (KapB), and kaposin C (KapC) are indicated with corresponding AUG, GUG, and CUG start codons for each. DR1, direct repeat 1; DR2, direct repeat 2; K12, the KapA ORF. (B) Example of the recoding strategy used to create the BAC16ΔKapB virus construct. Wild-type nucleotide and corresponding amino acid repeats are shown above with recoded nucleotides (red) and corresponding amino acid changes depicted below. (C) Lambda Red recombination method used to create all kaposin-deficient BAC16 viruses. The strategy for BAC16ΔKapB is depicted as an example. (D) Each kaposin-deficient BAC16 construct is shown relative to that of the wild-type *kaposin* locus in its genomic position (only reverse strand is shown for simplicity).

The kaposin transcript is polycistronic and can be translated into at least three polypeptides: KapA, KapB, and KapC (Fig. 1A) (45), though we and others have observed multiple banding patterns on immunoblots that suggest additional translation products may also be derived from this locus (45, 46). Translation of KapA is initiated at a canonical AUG start codon located distal to the 5′ end of the transcript and encodes a small membrane spanning protein (47–49). KapC is translated in the same reading frame as KapA, making its carboxy-terminal region identical to KapA. However, translation of KapC is initiated at one of two noncanonical CUG start codons proximal to the 5′ end of the transcript. Therefore, in addition to the KapA open reading frame (ORF), the KapC translation product contains two 23-amino-acid direct repeat (DR) regions termed DR1 and DR2 (DR1, PSSPGTWCPPPREPGALLPGNLV; DR2, APQEPGAAHPRNPARRTPGTRRG) which are derived from three sets of GC-rich 23-nucleotide repeats (Fig. 1A and B). Translation of KapB is initiated at one of two possible noncanonical CUG or GUG start codons that are most proximal to the 5′ end of the kaposin mRNA but uses an alternative reading frame to that of KapA/C. The resulting polypeptide is comprised largely of the same two 23-amino-acid DRs (DR1 and DR2) found in KapC (Fig. 1B) but lacks the C-terminal membrane spanning domain found in both KapA and KapC. Moreover, different isolates of KSHV display expansion or contraction of both direct repeats, the functional significance of which is unknown; yet, regardless of the diversity in size, the core DR1 and DR2 repeats remain constant (45).

Our knowledge about individual kaposin protein function is derived largely from ectopic expression of individual kaposin proteins. KapA was reported to promote cell transformation *in vitro* and *in vivo* (47, 48); however, more recent work showed that this function stems from one of the viral miRNAs embedded within the KapA ORF (50). Previous work from our group has shown that KapB is sufficient to induce angiogenesis in an *in vitro* EC tubule formation assay and to promote actin stress fibers and elongation of ECs into a spindled morphology that recapitulates the morphology of latently infected KS tumor cells (51). KapB also is sufficient to induce the disassembly of RNA granules called processing bodies (PBs) (51, 52). PBs are important sites for the constitutive turnover or translational suppression of inflammatory cytokine mRNAs; PB loss correlates with enhanced levels of inflammatory cytokine transcripts that are normally targeted to PBs via an AU-rich destabilizing element (51, 53–57). However, our previous studies could not determine whether KapB alone is necessary for these phenotypes during KSHV latent infection as we used RNA silencing to knockdown the kaposin transcript, a strategy that reduced expression of all kaposin proteins (51). No other studies have determined the role of individual kaposin translation products in KSHV latent or lytic replication cycles.

Here, we describe our use of the KSHV BAC16 bacterial artificial chromosome (BAC) and lambda Red recombination system (58) to construct a panel of viruses defective for specific *kaposin* locus products. We have constructed a triple deletion BAC16ΔKapABC, a double deletion BAC16ΔKapBC, and two single deletions BAC16ΔKapB and BAC16ΔKapC (Fig. 1C). We confirmed that these viruses no longer expressed the deleted kaposin-derived proteins and showed that all kaposin-deficient viruses were competent for genome replication and production of progeny virions. However, all kaposin-deficient viruses were significantly impaired when used to infect naive primary ECs, characterized by diminished genome copies after infection. ΔKapB was the most severely impaired, with markedly reduced genome copy number, small LANA nuclear bodies, and lower LANA expression, but none of these defects were complemented by providing KapB in *trans*. In addition, ΔKapB failed to cause PB disassembly following *de novo* infection of primary ECs, showing for the first time that KapB is necessary for PB disassembly during KSHV latency. With this panel of kaposin recombinant KSHV viruses, it is now feasible to interrogate, with unprecedented precision, the role of individual kaposin protein products in the context of KSHV latent or lytic infection.

## RESULTS

### Construction of a panel of kaposin recombinant BAC16 KSHV viruses.

Work on the *kaposin* locus has been limited by the lack of recombinant viruses that target this region. To address this limitation, we created a panel of recombinant viruses that delete KapA, KapB, and KapC individually or in combination. These recombinant viruses were created using the KSHV bacterial artificial chromosome clone BAC16, which contains the pBelo45 plasmid inserted between the vIRF-1 and ORF57 genes of the JSC-1 KSHV isolate (58, 59). This insertion encodes a red fluorescent protein (RFP) and a hygromycin resistance gene under the control of a constitutive cellular promoter to enable visual and chemical selection of BAC16-containing cells, respectively. The wild-type (WT) kaposin mRNA encodes three ORFs: KapA, KapB, and KapC (Fig. 1A). As a first step to create viruses that no longer made one or more of these protein products, we removed the DR regions that encompass the entire KapB ORF and most of the KapC ORF; in so doing, we created BAC16ΔKapBC. We then mutated the KapA start codon in the context of BAC16ΔKapBC to create BAC16ΔKapABC. However, creating single deletions of KapB and KapC by simple start codon mutagenesis proved to be extremely difficult due to the GC-rich and repetitive nature of this region. We decided to use an amino acid recoding strategy to construct BAC16 genomes that failed to code for either KapB or KapC, but not both. While both ORFs are comprised largely of 23-nucleotide DRs that translate into 23-amino acid DRs, the ORFs are in different reading frames. Using the BAC16 genome (accession GQ994935), we designed two different gene blocks with the nucleotide sequence of the DRs recoded. The first DNA construct was composed of alternative codons that disrupted the protein coding capacity of the KapB ORF, due to several nonsynonymous or nonsense mutations in the DRs, but contained only synonymous nucleotide changes in the KapC ORF that did not disrupt its amino acid sequence (ΔKapB; Fig. 1B). The second gene block coded for the inverse mutant; DRs were recoded such that the KapC ORF contained nonsynonymous or nonsense mutations, while the KapB ORF contained synonymous mutations that did not disrupt its amino acid sequence (ΔKapC; Fig. 1B). This strategy also drastically decreased the GC-content of DR region from 78 to 59.6% or 63.3% for ΔKapB and ΔKapC, respectively, facilitating the Lambda Red-based recombination events that followed and enabling us to use colony PCR screening during mutagenesis.

**FIG 2:**
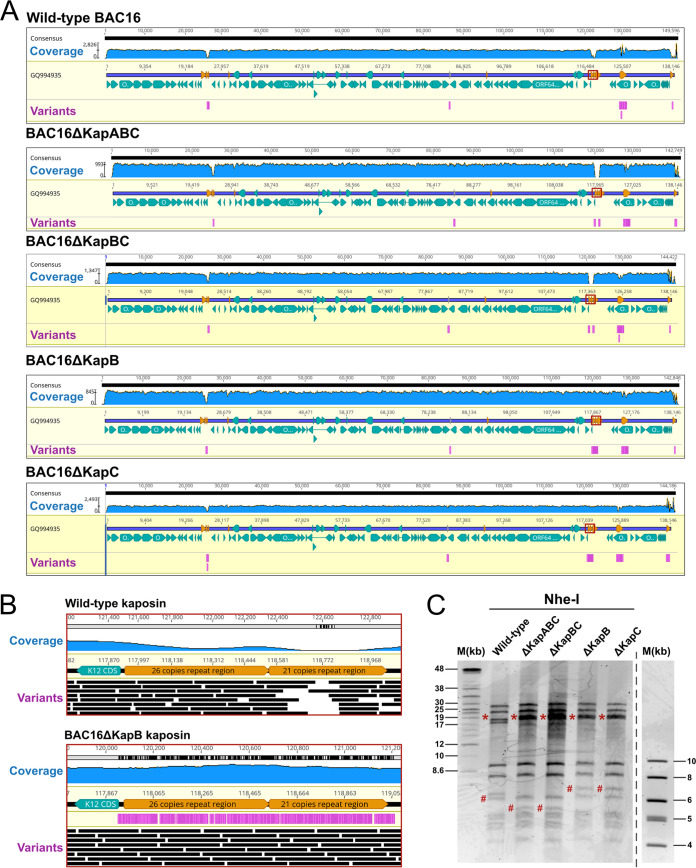
Confirmation of the panel BAC16 kaposin-deficient viruses. (A) BAC16 DNA was isolated from GS1783 *E. coli* harboring recombinant or WT BAC16 DNA and then subjected to whole-genome sequencing (WGS). Raw reads were paired, trimmed, and mapped to the BAC16 genome (NCBI accession GQ994935) in Geneious Prime. Coverage for each recombinant is indicated in blue with an average coverage per nucleotide of between 670× and 1,430×. BAC16 ORFs are depicted in turquoise, repeat regions are shown in orange, and the *kaposin* locus is outlined by a red box. Variants were detected based on a minimum coverage of 5 and a minimum frequency variation of 0.25 and are depicted in magenta. (B) Representative zoom panels of read alignment to the *kaposin* locus of WT and ΔKapB. Black rectangles depict individual reads aligned to this region. (C) BAC16 DNA was isolated from GS1783 *E. coli* and digested using NheI, and fragments were subjected to pulsed-field gel electrophoresis (PFGE). Based on *in silico* predictions, *kaposin* locus containing fragments are indicated with a hash symbol, whereas TR-containing fragments are indicated with an asterisk. Two different DNA ladders were included on each side of the gel to aid estimation of fragment sizes. The 4- to 10-kb DNA ladder was distal to the sample set; therefore, it is shown as a separate image from the main gel image.

A two-step lambda Red-based recombination method in *E. coli* was used to facilitate BAC16 mutagenesis (Fig. 1C), as reported previously (60). We isolated each recombinant BACmid from *E. coli* and performed whole-genome sequencing (WGS). Mapping of reads to the BAC16 reference genome (NCBI accession GQ994935) using Geneious Prime (61) revealed an average coverage per nucleotide of between 670× and 1,430×. Regions of bacmid DNA that were altered from the reference genome are termed variants; these regions were detected based on a minimum coverage of 5 and minimum frequency variation of 0.25 and are depicted in magenta. Based on this method of detection, no off-target mutations had occurred during recombination (Fig. 2A). Few variants were observed and these occurred within repetitive regions of the KSHV genome that also displayed low coverage, as previously shown (62–64). Outside of these, the only other variants detected were in the region between the genes for vIRF-1 and ORF57 (Fig. 2A). This is to be expected, since this is where the pBelo45 plasmid was inserted to facilitate the creation of BAC16, thereby altering the wild-type nucleotide sequence in this genomic location (58). We focused our attention to the kaposin DRs in WT BAC16. The repetitive nature of this sequence makes it difficult to resolve by WGS (65) and we observed low coverage. There was no coverage in the DR region for both ΔKapABC and ΔKapBC constructs, consistent with the complete removal of the DRs (Fig. 2A). In contrast, we observed high read coverage of the recoded kaposin regions in ΔKapB and ΔKapC viruses as recoding decreased the repetitive and GC-rich nature of the DRs (Fig. 2B; only WT and ΔKapB are shown for simplicity). The consensus sequences produced for these regions matched our recoded gene blocks, reaffirming successful recombination. Given the difficulty in resolving DR regions through Illumina sequencing (65), we also checked the BAC16 genomes using restriction fragment length polymorphism. BAC16 DNA isolated from *E. coli* was digested using the restriction enzyme NheI, followed by pulsed-field gel electrophoresis (PFGE) to resolve fragments. We observed fragment sizes consistent with predictions from *in silico* analysis using NCBI accession GQ994935 except for the fragment comprised largely of 801-bp terminal repeats (TRs). We predicted that the TR-containing fragment should migrate at 33 kbp; however, we did not observe a fragment of this size and instead observed an unexpected fragment migrating at approximately 18 kbp. This suggested a deletion of ∼15 kbp, or 19 copies of the 801-bp sequence, within this region. This deletion has previously been observed in BAC16 (66) because the highly repetitive nature of the TRs, as well as their high GC content, renders them sensitive to recombination events (67, 68). The smaller TR fragment was found in WT BAC16 and in all five recombinant viruses, suggesting the event occurred prior to our mutagenesis, and that analysis of our panel kaposin-deficient BAC16 viruses relative to WT will be unaffected. PFGE analysis also revealed a second size discrepancy within the predicted kaposin-containing fragment which WGS was unable to resolve due to the repetitive nature of the region. Based on *in silico* analysis, we expected to observe a decrease of 1,200 bp in the size of our kaposin-containing fragment (from ∼6,300 bp to ∼5,100 bp) for both our ΔKapABC and ΔKapBC genomes, corresponding to the removal of the DRs fragment. However, the digestion of these genomes produced a smaller than predicted decrease of only ∼1,000 bp, resulting in the fragment migrating closer to 5,300 bp instead. This suggests that over time the WT BAC16 construct lost 200 bp, or approximately three 23-amino-acid repeats, within the DR region, making our recoded kaposin locus in ΔKapB and ΔKapC viruses longer than the kaposin locus in WT BAC16. Because different KSHV isolates possess variable numbers of repeats (45), it is not surprising to observe that this repetitive genomic area experienced DR contraction during BAC16 DNA copying, like has been observed for the *kaposin* locus in KSHV. In future studies, we hope to decipher whether there is any functional significance to the number of each kaposin DR1 and DR2 for KSHV replication.

### Generation of stable BAC16-iSLK.RTA cell lines.

**FIG 3:**
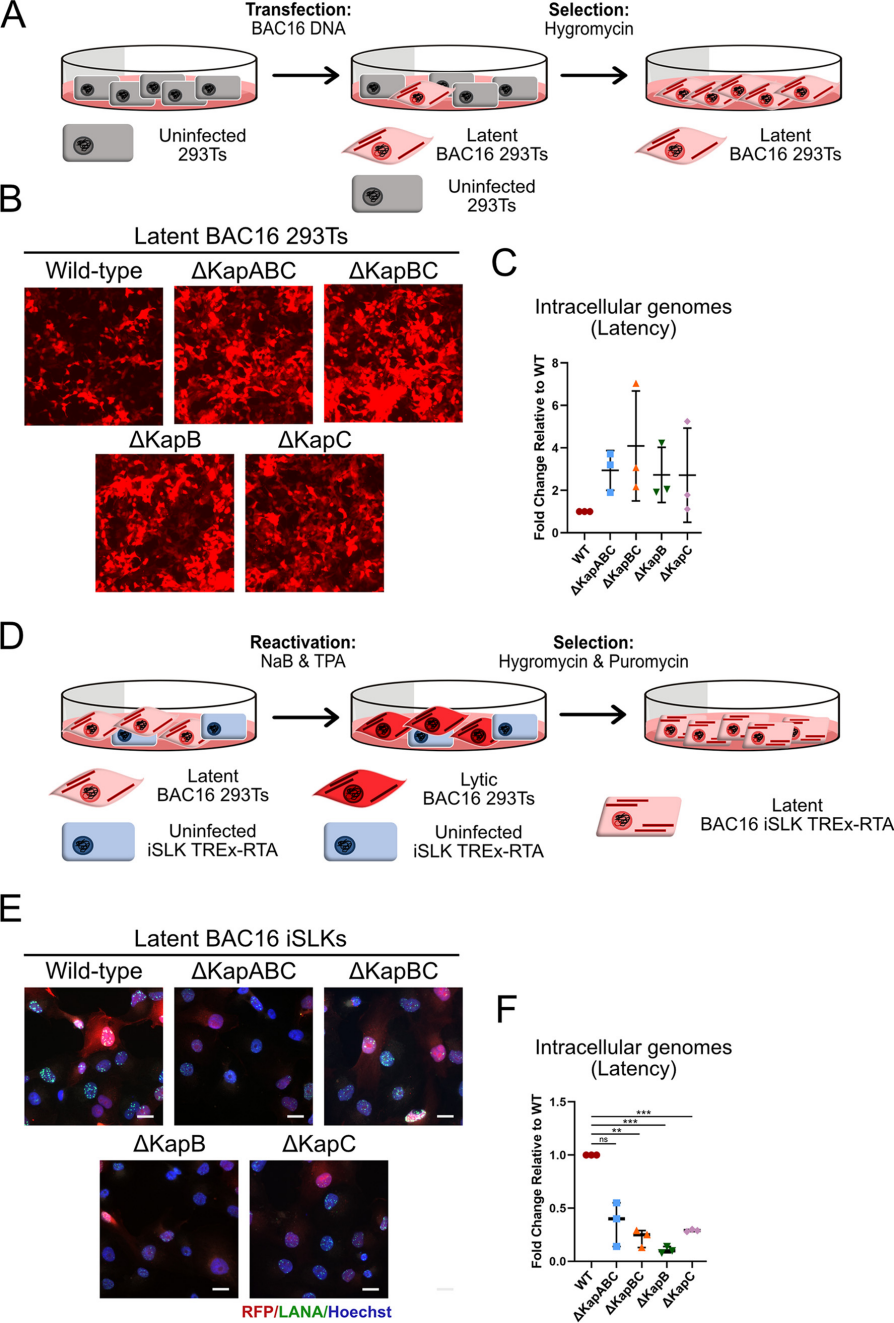
Generation of stable latent kaposin-deficient BAC16 iSLK cell lines by coculture method. (A to D) 293T cells were transfected with recombinant or WT BAC16 DNA. At 48 h posttransfection, cells were selected with hygromycin (increasing concentration from 50 to 100 μg/mL) until a confluent layer of BAC16-containing 293T cells was achieved. (B) Images of BAC16-293Ts were captured using an EVOS-FL microscope with the 10× objective. (C) Latent BAC16-293T cells were trypsinized, pelleted, and lysed to extract DNA. qPCR was performed using ORF26 and β-actin specific primers. Values are represented as the fold change relative WT. *n *=* *3; means ± the SD. (D to F) BAC16-293Ts were seeded in a 2:1 ratio with naive iSLK cells. Cells were treated with 1 mM NaB and 20 ng/mL TPA for 96 h, virus-containing supernatant was removed and replaced with medium containing puromycin (10 μg/mL) and hygromycin (increasing concentration from 200 to 1,200 μg/mL) until a confluent layer of BAC16-containing iSLK cells was achieved (E). Latent iSLKs were fixed, permeabilized, and immunostained using antisera for viral LANA (green) to visualize infected nuclei and Hoechst to visualize total cell nuclei (blue). Images were captured using Zeiss AxioObserver Z1 microscope with the 40× objective. Scale bar, 20 μm. (F) BAC16-containing iSLKs were trypsinized, pelleted, and lysed to extract DNA. qPCR was performed using ORF26 and β-actin specific primers. Values are represented as the fold change relative to WT. *n *=* *3; means ± the SD (*, *P < *0.05; **, *P < *0.01; ***, *P < *0.001; ****, *P < *0.0001; ns, nonsignificant).

After sequencing, the BAC16 mutant genomes were used to generate stable iSLK cell lines. A workhorse cell line for KSHV research, iSLK cells have been engineered to express the viral lytic switch protein, RTA, in a doxycycline (Dox)-inducible manner (69). These cells are especially useful for KSHV research as they maintain tight control of latency and produce large quantities of virus following the addition of Dox (69). However, iSLK cells were refractory to transfection in our hands; thus, we first transfected BAC16 DNA into 293T cells to create stable BAC16-293Ts for each BACmid (Fig. 3A), as reported previously (66). The efficiency of 293T transfection was between 20 and 30%, as estimated by RFP expression. We gradually selected the transfected cells with hygromycin until a confluent monolayer of RFP^+^ 293T cells was achieved (Fig. 3B). Via quantitative PCR (qPCR) for viral DNA (ORF 26), we analyzed intracellular genome copy number for each kaposin-deficient transfected 293T latent cell line and found that intracellular genome copy number was similar to WT in each case (Fig. 3C), consistent with the delivery of equal amounts of each bacmid DNA to 293Ts via transfection.

**FIG 4:**
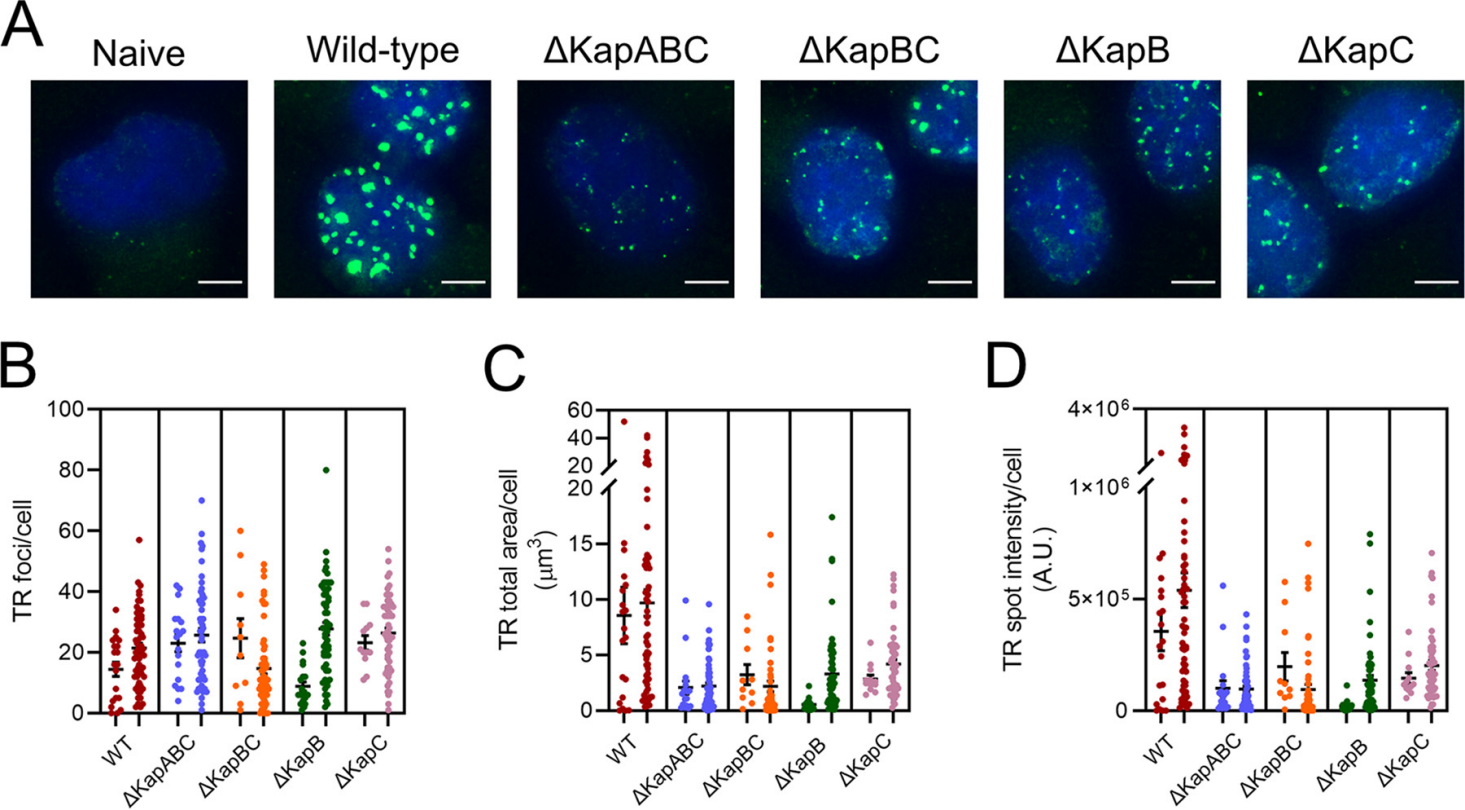
Kaposin-deficient latent iSLK cell lines display smaller genomic foci. WT or each kaposin-deficient latent iSLK cell line were seeded on coverslips, fixed, and stained for KSHV genomic DNA using Alexa Fluor 488-conjugated PNA probes specific for sequences in the TRs. (A) Representative wide-field immunofluorescence images of each latent iSLK cell line. PNA staining (green) and DAPI staining (blue) were performed. Images were captured using Nikon Eclipse Ti inverted epifluorescence microscope using a 60×/1.4 NA oil objective. Scale bar, 5 μm. Using ImageJ, the number of genomic foci per nucleus (B), the total area of TR per cell (C), or the total intensity of TR per cell (D) was quantified. *n *=* *2; quantified >50 cells per *n*; means ± the standard errors of the mean (SEM).

BAC16-containing 293T cells were then cocultured with naive iSLKs and reactivated using a histone deacetylase inhibitor (NaB) and a protein kinase C agonist (TPA) to produce progeny virions to enable primary infection of iSLKs (70–73). All kaposin-deficient viruses were able to infect naive iSLKs judging by RFP expression. The population of kaposin-deficient latent iSLK cells that was established by this coculture and infection procedure for each recombinant was then expanded via gradual selection in the presence of hygromycin to eliminate uninfected cells and puromycin to eliminate BAC16-293T cells, as iSLKs are puromycin resistant, until a confluent layer of RFP-positive cells was achieved (Fig. 3D). Immediately after selection, we analyzed the features of our iSLK latent cell lines. RFP-positive iSLK cells were then stained by immunofluorescence to visualize the viral latent protein LANA, which forms nuclear puncta called LANA nuclear bodies (NBs) during KSHV infection (9–12) that were visible in all our kaposin-deficient latent iSLK cell lines (Fig. 3E), suggesting latency was successfully established. We observed that not all LANA-positive cells were RFP positive, a discrepancy that has been reported by others (74). We also observed variable RFP intensity in our latent cell populations and an overall decreased intensity of RFP signal in kaposin-deficient cell lines relative to the WT (Fig. 3E). Using qPCR to quantify intracellular genome copy number of our panel of latent iSLKs, we revealed that kaposin-deficient iSLKs contained 10 to 40% of the genome copies observed in latent WT iSLKs (Fig. 3F). This was in stark contrast to the approximately equal amount of KSHV DNA copies observed between different populations of latent 293T cells that were created by transfection. This observation suggests the possibility that KSHV recombinant viruses that lack the *kaposin* locus display defects in their ability to establish latency and/or undergo genome amplification immediately following *de novo* infection, a step that was required to create latent iSLKs and that would normally serve to increase latent episome copy number (6, 7). To confirm genome copy number decreases in each of the kaposin-deficient latent iSLKs using a single cell method, we performed fluorescent *in situ* hybridization (FISH) for the KSHV genome. For this method, we used Alexa Fluor 488-conjugated PNA probes that hybridized with the TRs of KSHV, resulting in a fluorescent signal that is proportional to the amount of KSHV DNA per cell. We observed numerous large genomic foci in WT latent iSLK cells (Fig. 4A). In comparison, all kaposin-deficient latent cell lines displayed genomic foci of smaller area and lower intensity (Fig. 4A). To quantitatively compare these samples, we performed image analysis on these foci using ImageJ software to assess focal number, area, and intensity (Fig. 4B to D). In the data presented, each dot represents the total quantified KSHV DNA per individual cell, and each column of dots represents an independent experiment. We noted that although there was no remarkable difference in the number of genomic foci per cell between WT and the kaposin-deficient cell lines (Fig. 4B), there was a marked decrease in the average size and average intensity of these foci for all kaposin-deficient iSLK cells relative to WT (Fig. 4C and D). Because the probes we used are complementary to known sequences within each TR, and we verified that our WT BAC16 virus contains the same number of TRs as each kaposin-deficient construct (Fig. 2C), we can conclude that the quantified reduction in TR area and intensity represents a decrease in the genome copy number per cell. These data are consistent with our analysis of genome copy number by qPCR and reinforce the observation that all four kaposin-deficient iSLK latent cell lines possess less KSHV episomes than WT latent iSLKs.

### Cells latently infected with kaposin-deficient viruses produce progeny virions when reactivated.

**FIG 5:**
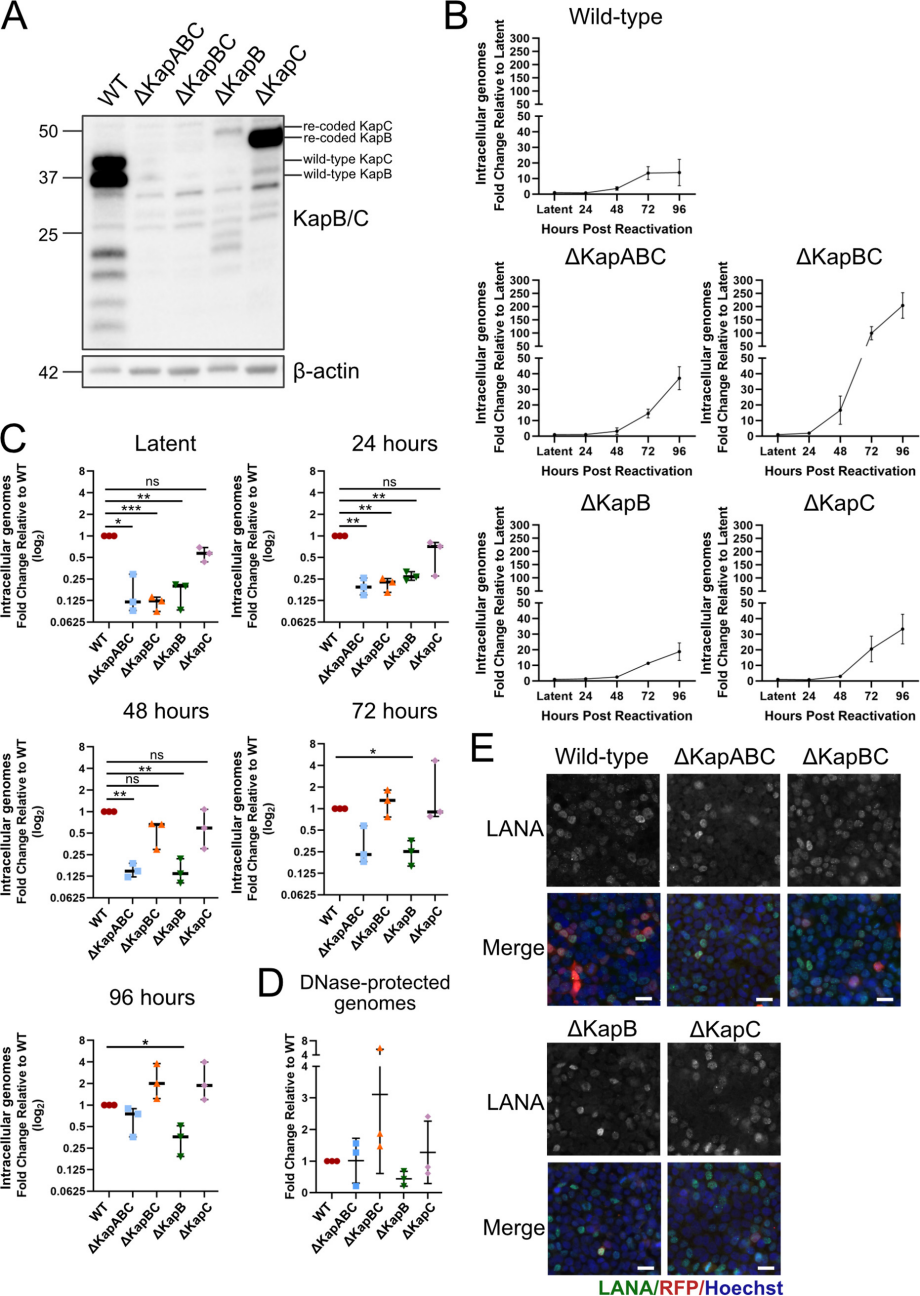
Kaposin-deficient BAC16 iSLK cells produce progeny virions when reactivated. (A) BAC16-iSLK cells were reactivated using 1 μg/mL Dox for 24 h prior to lysis and immunoblotting with antisera that recognizes DR1 in both KapB and KapC. The predicted bands representing KapB and KapC translation products are indicated. Specific antibody to β-actin served as a loading control. One representative experiment of three is shown. (B and C) BAC16-iSLK cells were reactivated with 1 μg/mL Dox and 1 mM NaB for 0, 24, 48, 72, or 96 h. At each time point, the cells were trypsinized, pelleted, and lysed to extract DNA. qPCR was performed using ORF26 and host β-actin specific primers. Values represent either the fold change relative to time zero (latency) for each recombinant virus (B; *n *=* *3, means ± the SD) or the fold change relative to WT at 0, 24, 48, 72, and 96 h postreactivation (C; *n *=* *3; means ± the SD; *, *P < *0.05; **, *P < *0.01; ***, *P < *0.001; ****, *P < *0.0001; ns, nonsignificant). (D) BAC16-iSLK cells were reactivated as described above for 96 h, and virus-containing supernatant was collected, clarified, and DNase treated prior to extraction of virion-protected DNA. qPCR was performed using ORF26 and luciferase (control) specific primers. Values represented the fold change relative to WT (*n *=* *3; means ± the SD; *, *P < *0.05; **, *P < *0.01; ***, *P < *0.001; ****, *P < *0.0001; ns, nonsignificant). (E) BAC16-iSLK cells were reactivated for 96 h. Virus-containing supernatant for each recombinant cell line was then collected, clarified, and diluted 1:4 with fresh DMEM and used to infect naive 293T cells. After 24 h, the cells were fixed, permeabilized, and stained using antisera specific for viral LANA (green) and Hoechst (nucleus, blue). Images were captured using Zeiss AxioObserver Z1 microscope with the 40× objective. Scale bar, 20 μm. The results from one representative experiment are shown.

Having created latently infected iSLK cell lines for each of our recombinant viruses, we confirmed the absence of KapB and/or KapC protein products after 24 h of reactivation of our ΔKapABC, ΔKapBC, and ΔKapB/ΔKapC latent iSLK cell lines by immunoblotting with a DR1-specific antibody (45) (Fig. 5A). Because both KapB and KapC may each utilize one of two noncanonical start codons, these proteins are comprised largely of the same DRs and are recognized by the same anti DR1 antibody, precise evaluation of kaposin translation products by immunoblotting is often difficult. WT-infected cells exhibited a range of DR-specific banding as has been previously observed, suggesting additional isoforms of KapB or KapC can be translated (45, 46). Predominant bands at approximately 41 and 35 kDa corresponded to the predicted molecular weights of full-length KapC and KapB, respectively. As expected, due to the absence of DR1 epitopes in these mutants, no KapB or KapC protein was detected in lysate from reactivated ΔKapABC or ΔKapBC iSLKs. We observed a faint band was detected at ∼46 kDa in lysate derived from ΔKapB-infected iSLKs which we think corresponds to the predicted molecular weight of recoded KapC (Fig. 5A). This version of KapC is larger than that encoded by our WT BAC16 because of the loss of DRs in our version of WT BAC16 (Fig. 2). A prominent band was consistently observed at ∼42 kDa in lysate from ΔKapC-infected iSLKs which is predicted to represent recoded KapB (Fig. 5A), also larger than WT-derived KapB for the same reason (Fig. 2).

To determine whether each of our kaposin-deficient iSLK cell lines was capable of reactivation, completion of a full lytic replication cycle, and the production of progeny virions, we reactivated the cell lines with Dox, to activate RTA expression, and NaB and measured intracellular genome copy number by qPCR at 24, 48, 72, and 96 h postreactivation. Genome copy number at each time point was normalized to its matched starting genome copy number in latency since each of the latent cell lines had a different episome copy number (Fig. 3F) prior to reactivation. For all recombinants, viral genome copy number significantly increased between 48 and 72 h after reactivation (Fig. 5B). WT and ΔKapB viruses exhibited ∼15-fold increase in intracellular genome copy number over latent copy number, ΔKapABC and ΔKapC viruses demonstrated an ∼40-fold increase, and ΔKapBC virus genome copy number increased ∼200-fold by 96 h post reactivation. We then determined how the reactivation process altered viral genome copy number for each kaposin-deficient recombinant relative to WT and observed that all kaposin-deficient viruses except for ΔKapB reached the same relative genome copy number as WT by 96 h postreactivation despite starting with fewer genomes than WT (Fig. 5C). These data show that all kaposin-deficient viruses are capable of genome replication to increase viral DNA copy number after reactivation. Because the starting point of the reactivation process was unequal (WT latent iSLK cells have more viral episomes at the ‘starting point’ of reactivation than any of the kaposin-deficient iSLK cell lines, including ΔKapBC latent iSLK cells, Fig. 3F), we were hesitant to draw quantitative conclusions about the efficiency of genome replication after reactivation between our panel of kaposin-deficient viruses.

We next sought to determine whether kaposin-deficient reactivated iSLK cells released progeny virus particles into the media. To do so, we harvested the virus-containing supernatant at 96 h postreactivation and subjected it to DNase digestion to eliminate unencapsidated viral DNA and then extracted the capsid-protected DNA and conducted qPCR for viral DNA (Fig. 5D). We found that kaposin-deficient viruses released similar numbers of DNase-protected viral genomes to WT (Fig. 5D). Although this observation suggests that the ability to package genomic material and assemble and release progeny virus does not require the *kaposin* locus, we and others have observed that extracellular genome copy number does not always correlate with infectious virus (21). To determine whether released virus from each kaposin-deficient latent iSLK cell line were competent to infect naive cells, we incubated a naive monolayer of 293T cells with supernatant from reactivated iSLK cells and, 24 h later, stained cells for the latent protein LANA. We identified LANA-expressing cells in all 293T cell monolayers infected by the panel of kaposin-deficient recombinant viruses (Fig. 5E) showing that each kaposin-deficient virus can produce infectious progeny virions. This is consistent with our earlier observations using the 293T-iSLK coculture system where transfected 293Ts produce virions that infect naive iSLKs to create latent cell lines, a method that relies on successful lytic replication and virion production.

### Latent iSLK cells that lack kaposin B display altered latency phenotypes.

**FIG 6:**
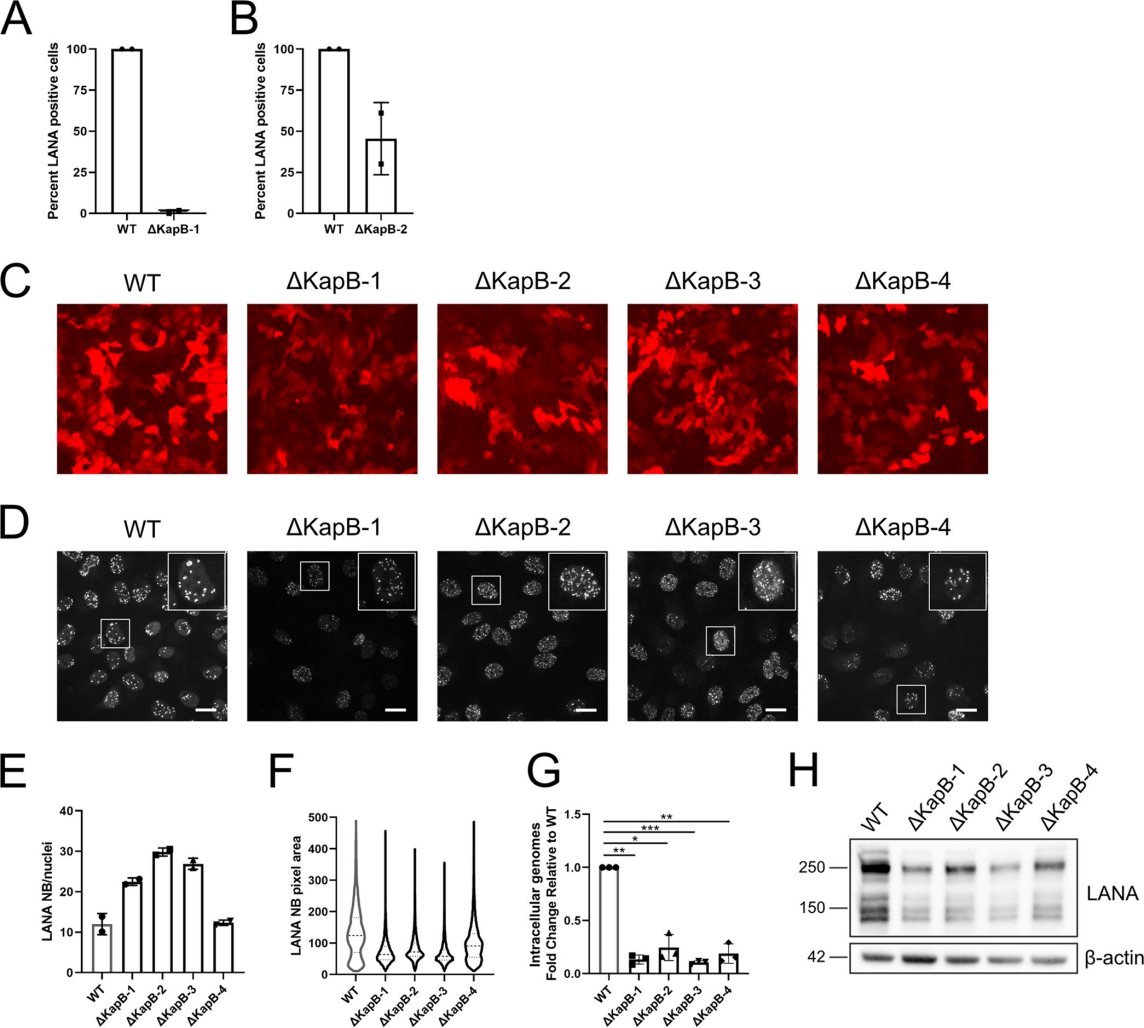
Primary infection of endothelial cells with BAC16ΔKapB virus is impaired. (A and B) Equal numbers of virus particles (based on DNase-protected extracellular genome copy number) of WT and either ΔKapB-1 and ΔKapB-2 were used to infect HUVECs. After 24 h, the cells were fixed, permeabilized, and immunostained for viral LANA and then stained with Hoechst. The number of LANA-expressing cells was quantified and is represented as a percentage of the total number of cells counted. *n *=* *2; means ± the SD. (C) Images of four different ΔKapB BAC16-iSLK latent cell lines and WT BAC16-iSLKs were captured using an EVOS-FL microscope with a 10× objective. The results from one representative experiment of two are shown. (D to F) Latent ΔKapB and WT BAC16-iSLKs were fixed, permeabilized, and immunostained for viral LANA, then stained with Hoechst. LANA-NBs were visualized (D), and the number of LANA-NBs per cell was counted using CellProfiler (E). *n *=* *2; means ± the SD. LANA-NB area was calculated for approximately 4,000 individual LANA-NBs per cell line and the distribution of area shown in panel F. (G) Latent ΔKapB and WT BAC16-iSLKs were trypsinized, pelleted, and lysed to extract DNA. qPCR was performed using ORF26 and β-actin specific primers. Values are represented as fold change relative WT. *n *=* *3, means ± the SD (*, *P < *0.05; **, *P < *0.01; ***, *P < *0.001; ****, *P < *0.0001; ns, nonsignificant). (H) Latent BAC16-iSLKs were lysed and harvested for immunoblotting with LANA and β-actin specific antibodies. The results from one representative experiment of two are shown.

Previous work from our lab used ectopic overexpression of KapB to characterize its function independent of viral infection. Consequently, we were most interested in analyzing the behavior of the ΔΚapB recombinant virus. We reactivated WT and ΔKapB-BAC16 iSLKs to produce progeny virus, equalized extracellular genomic copy number, and infected naive cells for 24 h; however, we saw very few LANA-positive cells postinfection (Fig. 6A). Given the inconsistencies between stable BAC16 cell lines reported by others (75, 76), we decided to rederive another ΔKapB-latent iSLK cell line. Using the same population of ΔKapB-BAC16-transfected 293Ts cells and an identical protocol for coculture, selection, and expansion, we produced a new ΔKapB iSLK cell line that we will refer to as ΔKapB-2 iSLKs, whereas our original ΔKapB iSLK cell line will be referred to as ΔKapB-1. Following ΔKapB-2 iSLK reactivation, we noticed a greater cytopathic effect in reactivated ΔKapB-2 iSLKs than previously observed in reactivated ΔKapB-1 iSLKs. We then equalized the extracellular particle number as described above, infected naive cells with ΔKapB-2 BAC16, and observed a 2-fold reduction in LANA-positive cells compared to WT virus (Fig. 6B), a great improvement compared to ΔKapB-1. The large difference between infectious progeny production from ΔKapB-1 versus ΔKapB-2 latent iSLKs prompted us to derive two additional ΔKapB iSLK cell lines as described above, termed ΔKapB-3 and ΔKapB-4. We compared several features between these four ΔKapB iSLK cell lines. We noted that although all four latent cell lines were RFP positive, ΔKapB-1 latent iSLK cells did not fluoresce as brightly as WT or the other ΔKapB latent cell lines (Fig. 6C). We stained these latent cells for LANA nuclear bodies (NBs) and observed several differences between LANA NBs present in latent WT iSLKs compared to the four ΔKapB latent iSLK cell lines (Fig. 6D). Although WT latent cells had fewer LANA NBs per cell than all four ΔKapB latent cell lines (Fig. 6E), WT LANA NBs averaged a much larger area than in ΔKapB latent cell lines (Fig. 6F). Of the four ΔKapB latent cell lines, ΔKapB-4 displayed a LANA NB character that most closely resembled WT; namely, fewer LANA NBs were brighter and larger (Fig. 6D to F). However, all four ΔKapB latent cell lines showed episome copy numbers of ∼10 to ∼25% compared to that of WT latent iSLKs (Fig. 6G). Of these, ΔKapB-2 and ΔKapB-4 had higher genome copy numbers, consistent with ΔKapB-2 iSLK improved reactivation (ΔKapB-2, Fig. 6B) and ΔKapB-4 iSLK LANA NB character (ΔKapB-4, Fig. 6D to F). In addition, all four ΔKapB latent cell lines displayed reduced steady-state levels of LANA protein compared to WT (Fig. 6H). Taken together, these distinct differences clustered the four ΔKapB iSLK latent cell lines as defective compared to WT in terms of key latency phenotypes such as LANA NB area, steady-state levels of LANA protein, and KSHV genome copy number.

**FIG 7:**
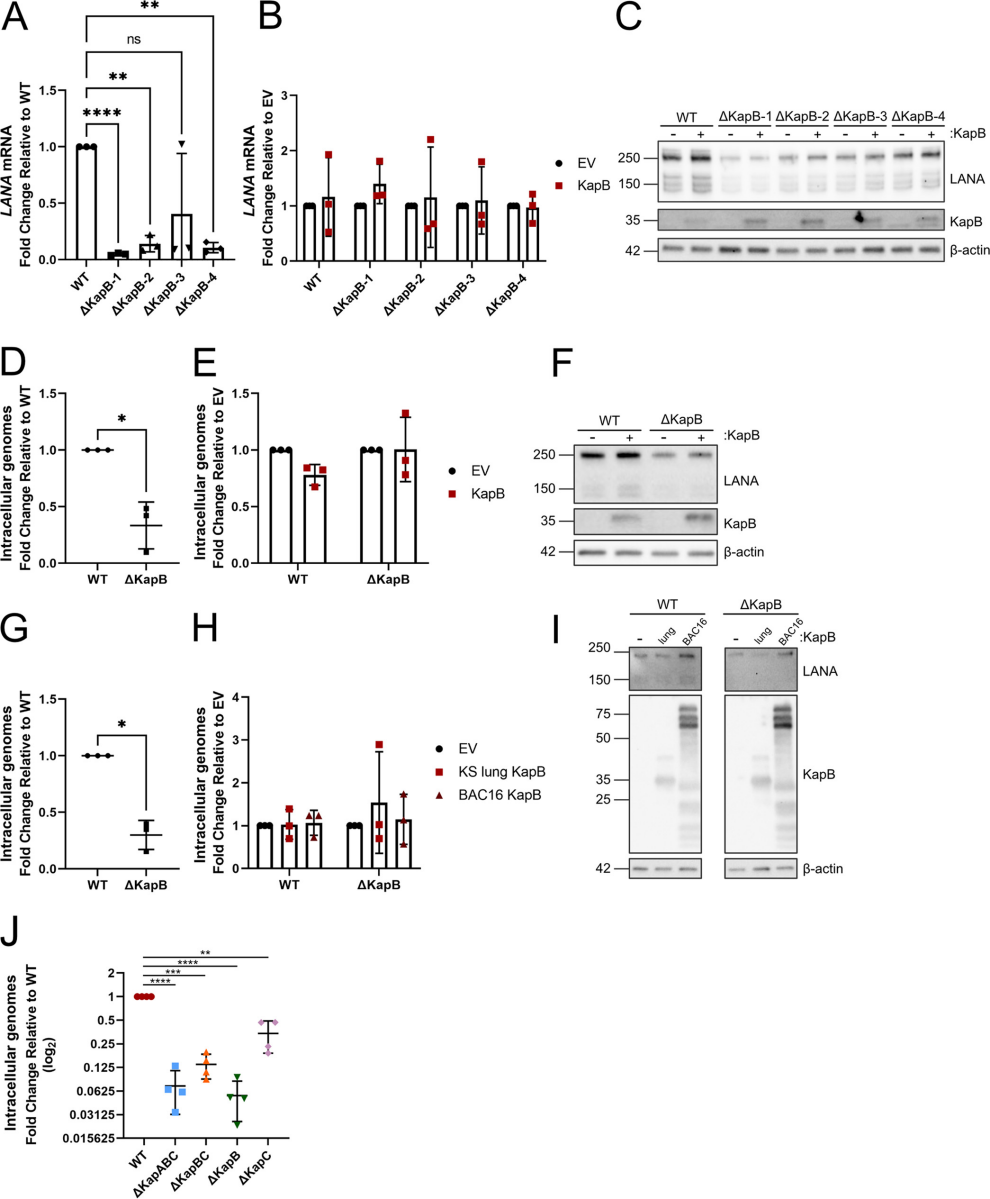
Ectopic expression of KapB does not rescue altered latency phenotypes in ΔKapB-infected cells. (A to C) Latent WT or ΔKapB BAC16-iSLK cells lines were transduced to express either an empty vector (EV) control or KapB (KS lung isolate). After 24 h, the cells were selected using blasticidin for 48 h prior to seeding. The following day seeded cells were lysed and harvested for RNA or protein analysis. (A and B) RT-qPCR was performed using LANA and 18S specific primers. *n *=* *3; means ± the SD (*, *P < *0.05; **, *P < *0.01; ***, *P < *0.001; ****, *P < *0.0001; ns, nonsignificant). In panel A, the LANA RNA transcript levels in ΔKapB iSLK cell lines are shown normalized to WT iSLK cells. In panel B, the LANA RNA transcript levels are shown after KapB complementation, normalized to each respective EV control. (C) Immunoblotting was performed using LANA, DR1 (KapB), and β-actin specific antibodies. The results from one representative experiment of three are shown. (D to F) Naive iSLK cells were transduced with EV or KapB lentiviruses as in panel A. After seeding, cells were infected with equal particle numbers of WT and ΔKapB BAC16 viruses, as determined via DNase-protected extracellular genome copy number. At 24 h postinfection, the cells were either trypsinized, pelleted, and lysed to extract DNA or lysed for immunoblotting. (D and E) qPCR was performed for KSHV DNA using ORF26 and host β-actin specific primers. *n *=* *3; means ± the SD. Statistical results were determined using a paired *t* test (*, *P < *0.05; **, *P < *0.01; ***, *P < *0.001; ****, *P < *0.0001; ns, nonsignificant). In panel D, the viral genome copy number for iSLKs after *de novo* infection with ΔKapB is normalized to the WT control. In panel E, the viral genome copy number for each virus infection after complementing with KapB is normalized to each respective EV control. (F) Immunoblotting was performed using LANA, DR1 (KapB), and β-actin specific antibodies. The results from one representative experiment of three are shown. (G to I) 293T cells were transfected with two different ectopic expression constructs for KapB: KapB derived from KS lung (45) and KapB BAC16. At 24 h after transfection, the cells were infected with equal particle numbers of WT and ΔKapB BAC16 viruses and processed as described above for qPCR (G and H, *n *=* *3; means ± the SD, statistical data were determined using a paired *t* test [*, *P < *0.05; **, *P < *0.01; ***, *P < *0.001; ****, *P < *0.0001; ns, nonsignificant]) or immunoblotting (I, *n *=* *1). In panel G, the viral genome copy number for 293Ts after *de novo* infection with ΔKapB is normalized to the WT control. In panel H, the viral genome copy number for each virus infection after complementation with different versions of KapB is normalized to each respective EV control. (J) HUVECs were infected with equal particle numbers of each virus, as determined by DNase-protected extracellular genome copy number. At 96 h postinfection, the cells were trypsinized, pelleted, and lysed to extract DNA. qPCR was performed using ORF26 and β-actin specific primers. Values are represented as fold change relative to WT. *n *=* *3; means ± the SD (*, *P < *0.05; **, *P < *0.01; ***, *P < *0.001; ****, *P < *0.0001; ns, nonsignificant).

To determine whether we could restore the defects associated with LANA expression in the established ΔKapB iSLK cell lines, we used lentiviruses to ectopically express KapB or an empty vector control (Fig. 7A to C). We used RT-qPCR to measure LANA transcript levels with and without complementation. ΔKapB latent iSLK cell displayed ∼10 to ∼40% of the LANA RNA transcript levels detected in WT iSLKs (Fig. 7A). Overexpression of KapB failed to alter LANA transcript levels compared to the control in any of the ΔKapB iSLK cell lines (Fig. 7B). Moreover, latent ΔKapB iSLK cell lines showed reduced steady-state levels of LANA protein, as described above, that was again not restored by providing KapB in *trans* (Fig. 7C). To determine whether complementation with KapB prior to primary infection and latency establishment could restore these defects, we transduced naive iSLK cells with KapB-expressing or control lentiviruses prior to primary infection with ΔKapB. KapB expression did not complement the low genome copy number or reduced LANA protein level seen in ΔKapB-infected cells after primary infection (Fig. 7D to F). To test whether KapB could complement primary infection of another cell type, we transfected 293Ts with two different versions of KapB (KS lung and BAC16) prior to primary infection. Neither version of KapB was able to rescue low KSHV genome copy number or LANA protein levels after *de novo* infection of 293Ts (Fig. 7G to I). Taken together, these data suggest that an aspect of the kaposin locus is required in *cis*, since providing the KapB polypeptide in *trans*, via nontargeted lentiviral integration, cannot complement the defects associated with ΔKapB latency after primary infection.

### Kaposin-deficient viruses that do not express an authentic GC-rich kaposin transcript display diminished ability to establish a primary infection.

The observation that provision of KapB in *trans* was unable to restore defects associated with ΔKapB latent iSLK cells suggested that the defect of low genome copy number was not due to the loss of the KapB protein, or any one of the kaposin polypeptides, but rather due to another feature of the region. To determine whether all the kaposin-deficient BAC16 viruses were deficient, we equalized genome copy number for the four mutants in our panel and compared their ability to *de novo* infect HUVECs compared to an equal inoculum of WT BAC16 virus. We found that all kaposin-deficient viruses, those lacking the GC-rich repeat region (ΔKapBC and ΔKapABC) and those where one frame was recoded while retaining the other (ΔKapB and ΔKapC), showed significant reduction in genome copy number 96 h after primary infection of HUVECs, regardless of the specific protein eliminated (Fig. 7J). The common feature among the kaposin-deficient mutants is loss of the GC-rich repeats in the kaposin RNA transcript and DNA locus. The failure to complement defective ΔKapB latent cells and the consistency of the genome copy number defect across our entire panel suggest that a feature of the kaposin locus is required in *cis* to support primary infection.

**FIG 8:**
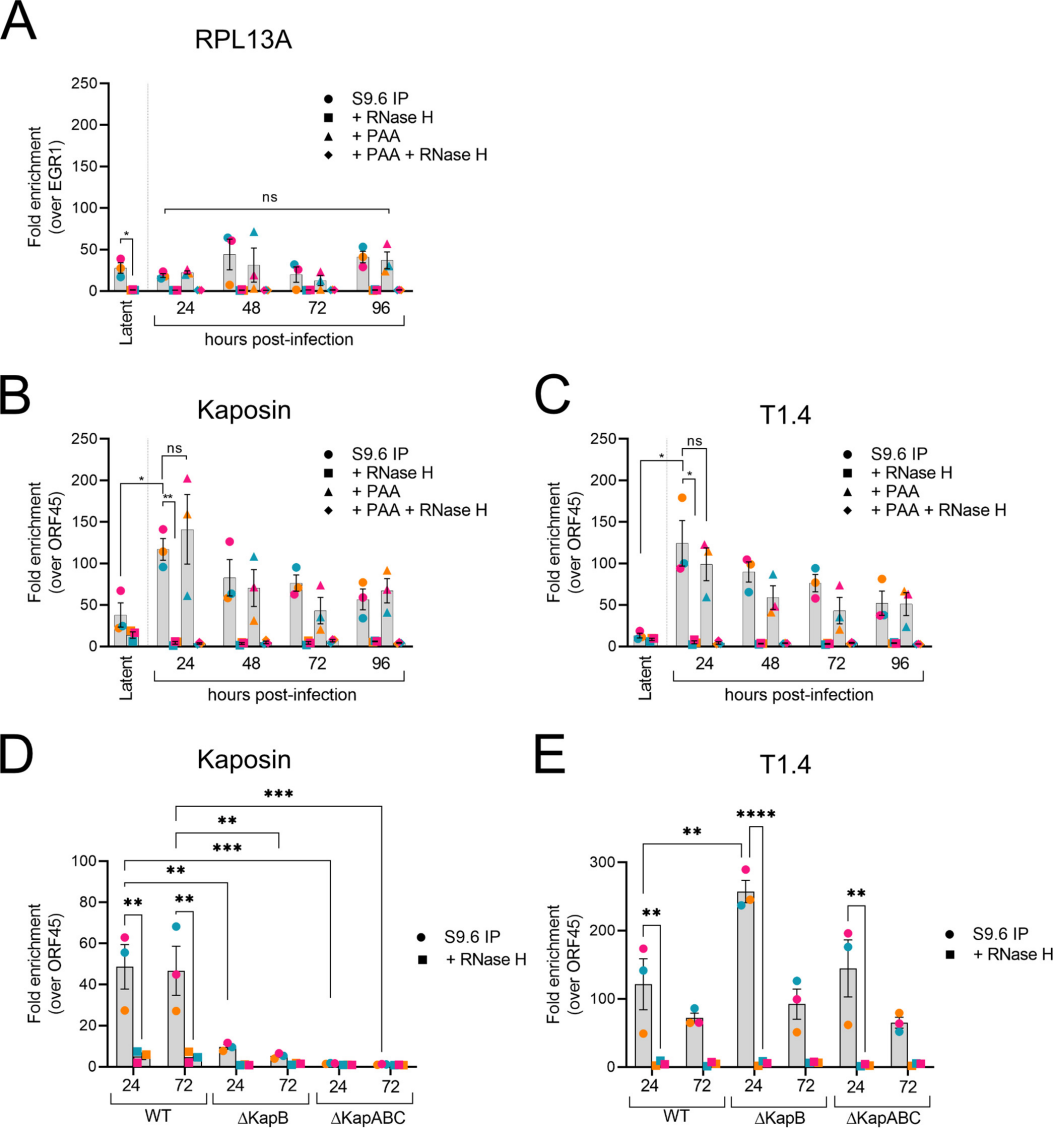
GC-rich kaposin repeats are required to form R-loops at the kaposin locus. WT BAC16-iSLK or selected kaposin-deficient iSLK cells were reactivated using 1 μg/mL Dox for 24, 48, 72, or 96 h prior to lysis, and a subset of samples were simultaneously treated with 500 μM PAA to prevent genome replication. Each lysate was then either treated or not treated with RNase H1 to degrade the RNA:DNA hybrid prior to immunoprecipitation with the monoclonal antibody S9.6. Precipitated DNA was analyzed by qPCR using specific primers for regions of cellular (A) or viral (B to E) DNA. (A) Positive control for R-loop formation in cellular DNA was the RPL13A locus. Values were plotted as the fold increase over the EGR1 human locus that does not form an R-loop. (B to E) Primer sets for the *kaposin* locus and T1.4 locus were used to determine and quantify the presence of RNA:DNA hybrids at each locus. Values were plotted as the fold increase over the ORF45 locus as a negative KSHV locus that does not form an R-loop (85). Statistical significance was determined using a two-way ANOVA with a Tukey’s *post hoc* test. *n *=* *3; means ± the SD (*, *P < *0.05; **, *P < *0.01; ***, *P < *0.001; ****, *P < *0.0001; ns, nonsignificant).

Both cytomegalovirus and Epstein-Barr virus have been shown to use an RNA transcript provided in *cis* to form a DNA:RNA hybrid structure called an R-loop at their respective origins of lytic replication, and in these studies this structure facilitated the recruitment of the viral DNA replication machinery (77–79). Given this, and our knowledge that R-loops preferentially form in GC-rich regions (80), we reasoned that if KSHV uses a similar strategy to promote genome replication, an R-loop would form at both KSHV lytic origin of replication, OriLytL and OriLytR. In line with this, others showed that the T1.4 RNA transcript derived from the GC-rich repeat region of OriLytL is required for viral genome replication at this origin specifically (81–83). However, no studies to date have considered the potential of the GC-rich kaposin transcript to be an important feature of the function of OriLytR, despite the proximal location of kaposin DRs to OriLytR (84). *In silico* analysis predicted that numerous R-loops would form in the KSHV genome; however, R-loop formation in KSHV DNA was only experimentally confirmed in the TR region (85). To experimentally determine whether R-loops form at both OriLyt regions in the KSHV genome, we performed an immunoprecipitation procedure for the DNA:RNA hybrids called DNA:RNA immunoprecipitation (DRIP) (77). In this procedure, we used the S9.6 monoclonal antibody to precipitate the hybrids from WT iSLKs and then performed qPCR for selected cellular and viral sequences to determine the presence of the RNA:DNA hybrids at specific loci. We first validated our procedure by pulling down a known R-loop forming sequence from the cellular gene RPL13A and plotted it as fold enrichment over the negative locus EGR1 (86) (Fig. 8A). To determine whether R-loops formed at the kaposin and T1.4 loci (part of OriLytR and OriLytL, respectively), we reactivated WT iSLK cells for the indicated times, and performed the DRIP procedure using primers to detect these viral sequences, ensuring that for the *kaposin* locus, we used primer sets that binds in the KapA region that remains intact in all the kaposin-deficient mutants. GC-rich sequences from both the T1.4 and kaposin transcripts were precipitated, suggesting that R-loops form at the T1.4 (OriLytL) and kaposin (OriLytR) loci in WT iSLK cells but not at ORF45 (a negative control as predicted earlier [85]) (Fig. 8B and C). To verify the authenticity of the pull downs for RNA:DNA hybrids, we treated lysates with RNase H1, an endonuclease that degrades the RNA moiety of the hybrid; and indeed, after RNase H1 treatment, these loci were no longer detected (Fig. 8A to C). We noticed that although both OriLyt regions were precipitated during latency, that this was significantly enhanced after reactivation, particularly for the kaposin region. To determine whether R-loop formation was influenced by prior DNA replication, we treated cells with phosphonoacetic acid (PAA) to inhibit viral DNA replication (4, 6, 87) and determined that PAA treatment had no effect on the precipitation of either locus (Fig. 8B and C) revealing that R-loop formation is independent from viral DNA replication.

Based on the low genome copy number of all our kaposin-deficient latent iSLK cell lines, and the fact that this defect was more pronounced after primary infection of ECs, we hypothesized that kaposin-deficient viruses that failed to transcribe an authentic GC-rich kaposin RNA would be unable to form an R-loop structure. When the *kaposin* locus was either deleted (ΔKapBC) or recoded such that the GC-rich character was reduced (ΔKapB), the kaposin region was no longer precipitated and did not contain any RNA:DNA hybrids (Fig. 8D). However, the T1.4/OriLytL R-loop remained and was even enhanced in the case of ΔKapB at 24 h postreactivation (Fig. 8E). These data show, for the first time, that KSHV forms R-loops at both origins of lytic replication and that mutations that destroy the GC-rich character of the kaposin transcript, a central feature of our panel of kaposin-deficient viruses, prevent R-loop formation.

### KapB is required for processing body disassembly after *de novo* infection of HUVECs.

**FIG 9:**
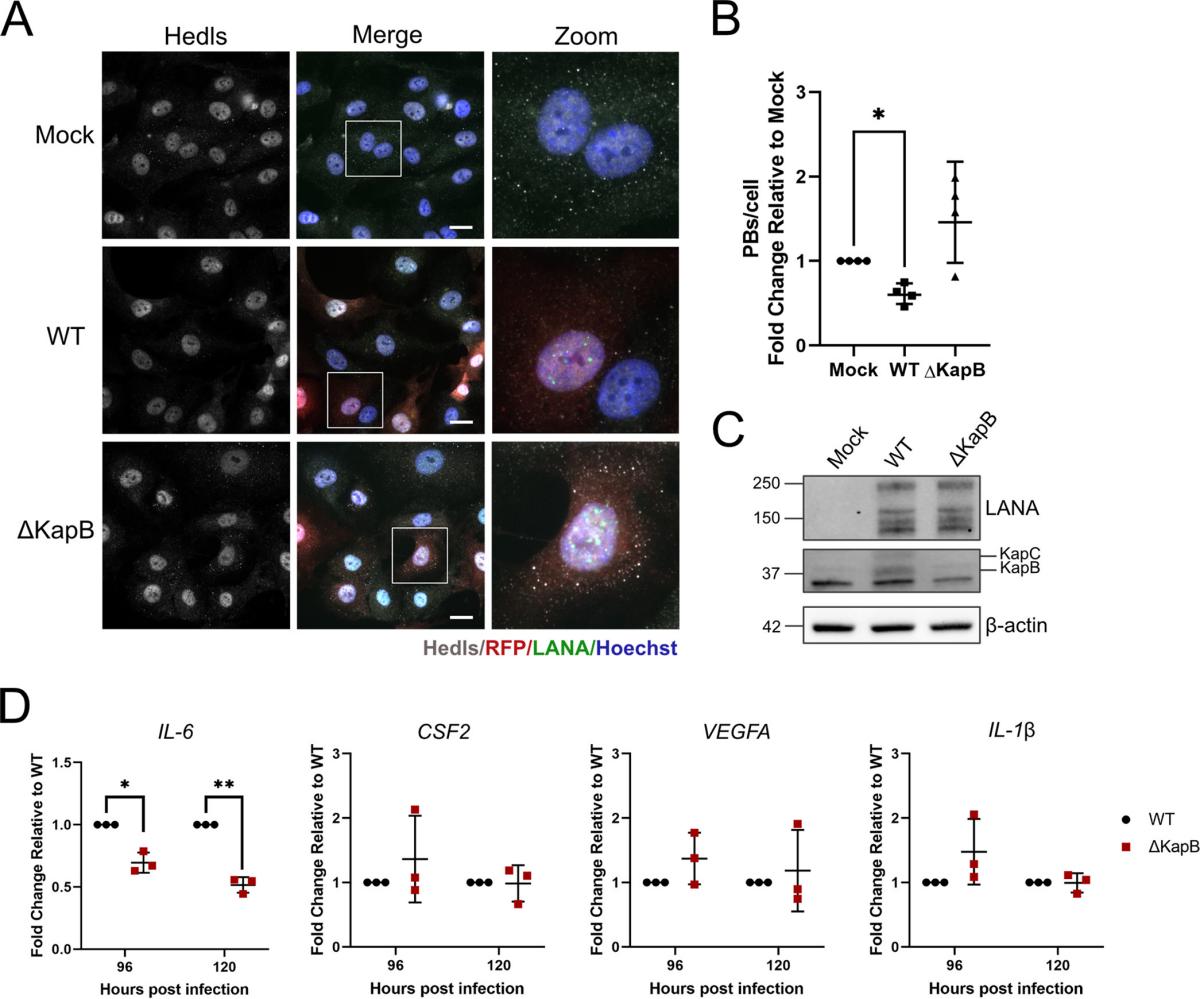
KapB is required for PB disassembly following *de novo* HUVEC infection. (A) WT and ΔKapB BAC16 viruses were titrated using 293T cells, and RFP expression was used to estimate an equal number of infectious virions for subsequent *de novo* infection of HUVECs. At 96 hpi, HUVECs were fixed, permeabilized, and immunostained for either viral LANA (green), the PB-resident protein Hedls/EDC4 (white), and then stained with Hoechst (nucleus, blue). Images were captured using Zeiss AxioObserver Z1 microscope with a 40× objective. Scale bar, 20 μm. (B) Hedls puncta per cell were quantified using CellProfiler. For mock infections, all cells were counted; for WT and ΔKapB-infected cells, only LANA-positive cells were counted. The data are represented as the fold change in PBs per cell relative to mock infection. *n *=* *4; means ± the SD (*, *P < *0.05; **, *P < *0.01; ***, *P < *0.001; ****, *P < *0.0001; ns, nonsignificant). (C) Cells infected as in panel A were lysed at 96 hpi and harvested for immunoblotting with LANA, DR1 (KapB and C), and β-actin specific antibodies. One representative experiment of three is shown. (D) Cells infected as in panel A were lysed at 96 and 120 hpi and harvested for RNA extraction and RT-qPCR using IL-6, CSF2, VEGFA, IL-1β, and HPRT specific primers. *n *=* *3; means ± the SD. Statistical data were determined using a paired *t* test (*, *P < *0.05; **, *P < *0.01; ***, *P < *0.001; ****, *P < *0.0001; ns, nonsignificant).

We have previously shown that ectopic expression of KapB in human umbilical vein endothelial cells (HUVECs) is sufficient to recapitulate key phenotypes associated with KSHV latency, including the disassembly of processing bodies (PBs), cytoplasmic RNA and protein granules whose loss correlates with enhanced inflammatory cytokine RNAs (51, 53–57). To determine whether KapB was necessary for KSHV-induced PB loss, we reactivated ΔKapB-2 latent iSLK cells and used this virus to infect HUVECs. After 96 h postinfection, we fixed the infected cells and stained the monolayer for both LANA and for a PB-resident protein called Hedls/EDC4 (88, 89). We observed that ∼80 to ∼90% of the cell monolayer was infected with either ΔKapB BAC16 or WT BAC16 virus. In LANA-positive cells that were infected with WT virus, we observed a 2-fold decrease in PBs per infected cell compared to the mock-infected control (Fig. 9A and B), as shown previously (51). In LANA-positive cells infected with ΔKapB-2 virus, PB numbers were not significantly altered relative to the mock-infected control (Fig. 9A and B). We also confirmed that WT-infected HUVECs expressed KapB, but that KapB was absent in ΔKapB-infected HUVECs (Fig. 9C). These data reveal for the first time that the KapB protein is not only sufficient but also necessary for KSHV-induced PB disassembly following *de novo* infection.

PBs constitutively repress or degrade cytokine transcripts that contain AU-rich elements (AREs); accordingly, we and others have observed that PB disassembly correlates with enhanced steady-state levels of some cytokine transcripts (51, 53–57). After primary infection of HUVECs with WT or ΔKapB, we measured steady-state levels of selected ARE-containing cytokine transcripts by RT-qPCR (Fig. 9D). We predicted that if PBs remain intact, as occurs after ΔKapB infection, cytokine levels would be decreased relative WT-infected cells as the ARE-RNAs from which they are expressed would be subject to PB-mediated decay. Consistent with this hypothesis, after infection with ΔKapB, IL-6 transcript levels were much reduced relative to that observed with WT infection (Fig. 9D). This is the expected response for a cytokine mRNA that is shuttled to PBs for constitutive decay. However, we did not observe a strong difference between steady-state levels of other ARE-containing cytokine transcripts (VEGF, IL-1β, and GM-CSF) after WT versus ΔKapB infection (Fig. 9D), since not all ARE-RNAs respond in the same manner to altered PB dynamics, as shown in a previous study (90). Moreover, IL-6 mRNA levels are extremely sensitive to changes in PBs (91), and we previously observed that IL-6 transcript levels increase in response PB disassembly induced by KapB (92). Taken together, these data show that KSHV-induced PB disassembly correlates with an ∼2- to 3-fold-increased steady-state level of the IL-6 transcript and that both PB disassembly and enhanced IL-6 RNA levels are absent in cells infected with ΔKapB.

**FIG 10:**
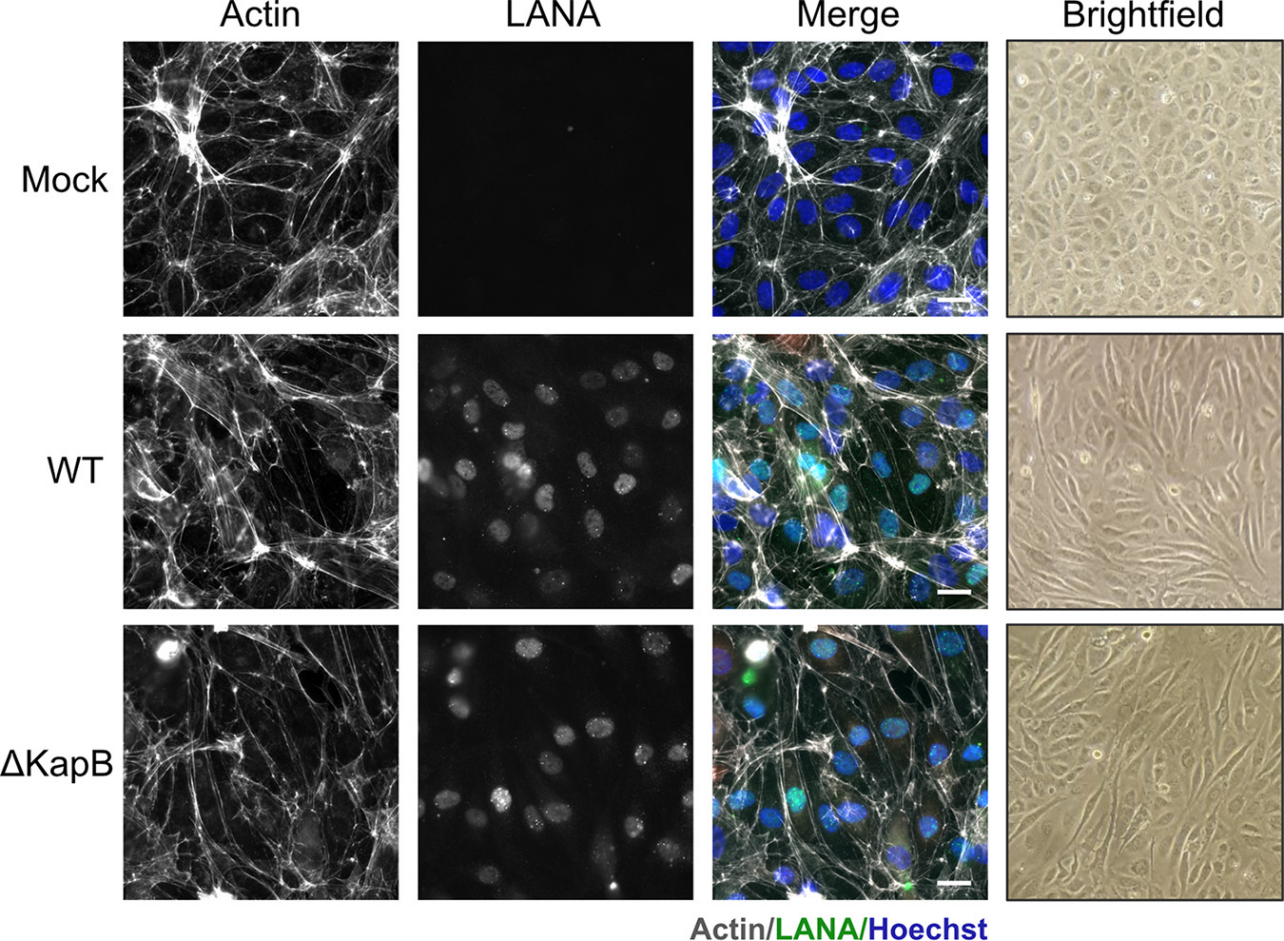
KapB is not required for endothelial cell spindling following *de novo* HUVEC infection. WT and ΔKapB BAC16 viruses were titrated using 293T cells, and RFP expression was used to estimate an equal number of infectious virions for subsequent *de novo* infection of HUVECs, as in Fig. 8. At 96 hpi, bright-field images were acquired at 10×, and the cells were fixed, permeabilized, and immunostained for viral LANA (green). Phalloidin and Hoechst staining was used to visualize actin (white) and nuclei (blue), respectively. Images were captured using Zeiss AxioObserver Z1 microscope with a 40× objective. Scale bar, 20 μm. The results from one representative image of three experiments are shown.

We previously showed that ectopic expression of KapB is sufficient to induce primary ECs to elongate and spindle, thereby recapitulating the morphology of infected cells within KS lesions (51, 93). However, others have observed that ectopic expression of v-FLIP likewise elicits EC spindling (94) and that the deletion of v-FLIP from the viral genome eliminates EC spindling, although this effect is overcome at high multiplicity of infection (95). To test whether KapB is necessary for EC spindling, we used WT of ΔKapB BAC16, derived as above, to infect HUVECs, fixed cells at 96 hpi and visualized spindling at low magnification by bright-field microscopy or at high magnification by staining fixed cells with phalloidin to label actin filaments. Robust cell spindling was observed in LANA-positive, WT-infected cells (Fig. 10). We also observed spindled cells in LANA-positive, ΔKapB-infected HUVECs (Fig. 10). We conclude that although ectopic expression of KapB is sufficient to elicit EC spindling (51), it is not necessary for EC spindling during KSHV latency.

## DISCUSSION

The kaposin locus comprises a significant portion of the coding capacity of the KSHV latency locus, yet its role in KSHV latent and lytic replication is not fully appreciated. Prior to this report, the KSHV field lacked recombinant viruses to enable functional analysis of individual kaposin proteins in the context of KSHV infection. Here, we described the construction and validation of a suite of kaposin-deficient recombinant viruses using the BAC16 bacterial artificial chromosome and a panel of corresponding stable iSLK cell lines. We deleted or recoded kaposin ORFs individually or in combination, generating several mutants: ΔKapABC, ΔKapBC, ΔKapB, and ΔKapC. We showed that all kaposin-deficient viruses established latency in iSLKs, replicated the viral genome upon reactivation, and produced progeny virions that could be used for *de novo* infection of naive cells. Despite this, all kaposin-deficient latent iSLK cells lines displayed reduced viral genome copy number during latency and after primary infection of ECs compared to WT. In addition, we characterized four derivations of ΔKapB-iSLKs and showed that these cells not only had markedly reduced genome copy number but also reduced LANA NB area and LANA RNA and protein levels. Attempts to complement the reduced expression of LANA in ΔKapB-iSLKs by ectopic expression of KapB had no effect, nor did attempts to complement the reduced KSHV genome copy number after primary infection with ΔKapB virus. Together, our data suggest that the kaposin locus plays a role in latency establishment that cannot be restored by complementing with KapB protein in *trans* and may involve the formation of an R-loop at the *kaposin* locus. Finally, we used ΔKapB BAC16 to show that KapB is not only sufficient but also necessary for KSHV-mediated PB disassembly following *de novo* infection of primary ECs, verifying the contribution of an individual kaposin protein to KSHV biology. This toolkit will allow us to understand the role of the kaposin locus in KSHV replication with unprecedented precision, making it a significant advance for the field.

Previous knowledge about the role of the kaposin locus was derived from studies using the ectopic expression of individual kaposin ORFs (47, 51, 52, 96–98). To date, no comprehensive kaposin-deficient BAC16 studies have been published. Toth et al., created a kaposin knockout BAC16 virus by deleting a 1,319-nucleotide region that included the coding region of KapA and the majority of the coding regions of KapB and KapC (99). This recombinant virus was used to test for the recruitment of proteins from polycomb repressive complexes 1 and 2 to two lytic viral promoters after primary infection of SLK cells (99). For this phenotype, the kaposin knockout virus displayed no difference from WT, but aside from this, no additional analyses related to this mutant were reported. Gallo et al. created a K12 mutant in which a kanamycin cassette disrupts the K12 ORF, but reported limited phenotypic analysis related to this virus (100). Finally, Campbell et al. investigated the role of the RTA-responsive element (RRE) promoter in the kaposin locus, and in so doing created a K12 RRE mutant; however, this study focused solely on RTA-recruitment and genomic architecture (101). In the present study, our recombinant viruses were created by removing all DR1 and DR2 sequences (ΔKapBC) with or without mutagenesis of the KapA ORF start codon (ΔKapABC) or by replacing the DR region with alternatively coded nucleotide sequences (ΔKapB and ΔKapC). Our recoding strategy had several advantages. First, the translation potential of the *kaposin* locus was reduced by insertion of multiple termination codons, eliminating expression of some protein products while maintaining other ORFs by alternative codon usage. Second, recoding destroyed the repetitive nature of the locus and decreased the GC content, making the region much more amenable to error-free homologous recombination. Third, in all the recombinant kaposin-mutant BACs, regulatory sequences for transcription and miRNA processing were left intact. We also created corresponding latent iSLK cell lines for the kaposin-deficient BAC16 constructs. All four of these kaposin-deficient latent iSLK cell lines showed drastic reductions in viral genome copy number compared to WT. The cell lines were created by coculturing BAC16-transfected HEK293Ts with naive iSLKs, a widely used method reported to be more efficient than direct transfection of recombinant BAC16 DNA, that leverages cell-to-cell spread of virions from reactivated 293T cells to iSLKs (5, 66, 69, 102). Despite using this strategy to select for bacmid-containing cells, we failed to produce latent cell lines that contained high genome copy numbers for any of the kaposin-deficient BAC16 DNA. The low KSHV episome copy number of each kaposin-deficient latent iSLK cell line was confirmed using FISH to stain for individual KSHV genomic foci. We then repeated the coculture procedure for the most impaired of these mutants four times, and each attempt yielded similar results; all four ΔKapB iSLK cell lines displayed reduced viral episome number and altered LANA NBs compared to WT BAC16 cells. Although these data are consistent with the idea that kaposin-deficient viruses are impaired in one or more key steps required for the establishment of latency, further study is required to characterize the nature of this defect.

LANA expression and function is central to the establishment of KSHV latency. LANA forms homo-oligomers and binds the terminal repeat (TR) regions of the KSHV genome while simultaneously binding histones H2A and H2B of host chromatin, thereby tethering the viral DNA to host chromosomes (8, 10, 11, 103). This binding leads to the formation of structures called LANA NBs that colocalize with viral genomes (10, 104, 105). The number of LANA-positive puncta in an infected cell was reported to correlate with the number of viral episomes (13). This is supported by evidence that KSHV *de novo* infection of a cell monolayer is heterogenous and results in variable levels of intracellular viral genomes between individual latent cells, and this heterogeneity is also represented by diverse LANA NB number per cell (13). Therefore, robust LANA NB staining is a suitable marker for successful latency establishment. However, we and others have observed heterogenous LANA NB size within and between latent infected cells, but the significance of this size variability is unclear. Because LANA can bind to nucleosomes in cellular chromosomes, as well as those bound to viral episomes (15), LANA NBs are classified as supramolecular structures that cluster multiple viral episomes together (12, 105). Genome clustering was proposed to be an effective evolutionary strategy that allows KSHV to rapidly increase viral genome copy number per cell at the expense of total number of cells infected (15). It is not yet clear whether a minimum number of viral episomes is required to create a supramolecular LANA NB after *de novo* infection; however, WT KSHV does preferentially form clustered LANA NBs (15). We have observed that latent ΔKapB iSLK cells possess smaller and more uniform LANA NBs compared to those found in WT iSLK cells and using FISH, showed that genomic foci were much smaller and dimmer in kaposin-deficient iSLKs than in WT iSLKs. Latent ΔKapB iSLK cells express smaller amounts of LANA RNA and protein, a phenotype that we could not complement by ectopic expression of KapB in *trans*. Therefore, we report an additional correlative phenotype between low genome copy number, smaller KSHV genomic foci, lower LANA expression, and smaller LANA NBs observed in ΔKapB latent iSLK cell lines and speculate that ΔKapB iSLK cells do not contain enough viral episomes and/or LANA protein to form large LANA NBs with clustered genomes.

The kaposin locus overlaps with one of two viral origins of lytic genome replication, called OriLytR. Though very few studies have investigated the contribution of OriLytR/kaposin to viral genome replication, detailed analyses on the function of OriLytL have revealed that it facilitates genome copying after viral reactivation and upon *de novo* infection (81–84). More recent work has shown an essential role for the RNA transcript derived from OriLytL (called T1.4) in viral genome replication after *de novo* infection (82). Other herpesviruses have been shown to use an RNA transcript derived from the lytic origin of replication to form an RNA:DNA hybrid called an R-loop to facilitate recruitment of the viral RNA replication machinery to the OriLyt (78, 79). Our work showed for the first time that R-loops formed at both lytic origins in the KSHV genome and that this structure failed to form at OriLytR when the kaposin GC-rich region was deleted (ΔKapBC) or recoded (ΔKapB) to alter its GC-rich repeated character. It is possible that the absence of the GC-rich kaposin repeat region and its complementary GC-rich RNA transcript in our mutants impairs the contribution of OriLytR to viral genome copying that occurs after *de novo* infection, as was observed for a recombinant virus that does not make the T1.4 transcript from OriLytL or for other herpesviruses (78, 79, 82). A reduction in viral genome copy number after *de novo* infection would be predicted to impair latency establishment and diminish the clustering of viral episomes into larger LANA NBs, which may explain the different LANA NB phenotype we observed in all four ΔKapB iSLK latent cell lines. Further experimentation will be needed to reveal more detail about the precise mechanism by which kaposin region may promote genome amplification after primary infection and how this alters LANA NB formation, function, and latency establishment.

KSHV latency predominates in most cells in culture and in KS tumors, suggesting viral proteins expressed at this time are important in promoting tumorigenesis. We have previously shown that ectopic expression of KapB in primary ECs recapitulates two features of the KS lesion: cell spindling and elevated inflammatory cytokine expression via PB disassembly. Using the ΔKapB BAC16 recombinant virus, we analyzed whether KapB was not only sufficient but necessary for these KSHV-associated tumor phenotypes. We show that after *de novo* infection of HUVECs, ΔKapB BAC16 cells can induce cell spindling to a similar extent as WT BAC16 virus. These data are consistent with reports that the viral latent gene vFLIP is also capable of causing endothelial cell spindling; therefore, the loss of KapB does not eliminate the spindling phenotype (94). We propose that KapB and v-FLIP may act synergistically to promote EC spindling during KSHV latency and that, in the absence of one, the other is able to compensate. We also used the ΔKapB BAC16 recombinant virus to determine whether KapB was necessary for KSHV-induced PB disassembly. PBs are cytoplasmic sites for the constitutive turnover or translational suppression of ARE-containing inflammatory cytokine mRNAs (51, 53–57). We previously showed that latent KSHV infection of primary ECs caused PB disassembly (51). Here, we also observed that 96 h after d*e novo* infection of HUVECs with WT BAC16, PBs were disassembled. However, d*e novo* infection of HUVECs with ΔKapB BAC16 did not cause PB disassembly, and steady-state levels of IL-6 RNA, an ARE-containing cytokine transcript known to be regulated by PB levels, were reduced relative to that after WT infection (91). This reveals that KapB is both necessary and sufficient for PB disassembly during KSHV latency and represents the first time that a phenotypic function can be specifically attributed to a single kaposin protein in the context of KSHV infection.

Our group and others showed that PBs also decrease during lytic replication and that more than one viral protein is sufficient for this effect (53, 106). Here, we observed PB disassembly 96 h after *de novo* infection of HUVECs, and we have previously shown PB disassembly in latent HUVECs (51). In contrast, Sharma et al. reported that PBs were not lost in a KSHV-infected latent primary effusion lymphoma cell line. This discrepancy could be attributed to the use of B-lymphocytes in their studies or their visualization of PBs by staining largely for the PB-resident protein GW182, an antigen that has been shown to adopt a punctate pattern distinct from other PB marker proteins (106, 107). Taken together, the observation that KSHV encodes multiple proteins in both phases of its replication to elicit PB disassembly suggests the functional relevance of this phenotype for viral infection. Because PBs constitutively repress many proinflammatory, angiogenic, or protumorigenic cellular transcripts, we propose that the absence of microscopically visible PBs may contribute to the inflammatory and angiogenic KS microenvironment and that KapB is necessary for this effect in KS lesions.

In summary, this work describes the construction and preliminary analysis of four different kaposin-deficient BAC16 viruses and corresponding iSLK latent cell lines. We used these mutants to specifically attribute the phenotype of PB disassembly following *de novo* infection of primary to the viral protein KapB. We hope these mutants will enable future studies to better evaluate the roles of individual kaposin protein products during infection. Moreover, we believe our strategy to recode protein products in multiple reading frames can serve as a mutagenesis roadmap for understanding other complex, polycistronic viral loci.

## MATERIALS AND METHODS

### Cell culture.

All cells were grown at 37°C with 5% CO_2_ and atmospheric O_2_. HEK293T cells (ATCC) were cultured in Dulbecco modified Eagle medium (DMEM; Thermo Fisher) supplemented with 100 U/mL penicillin, 100 μg/mL streptomycin, 2 mM l-glutamine (Thermo Fisher), and 10% fetal bovine serum (FBS; Thermo Fisher). iSLK.RTA cells (69) were cultured likewise with the addition of 1 μg/mL puromycin and 250 μg/mL Geneticin (Thermo Fisher). BAC16-iSLK.RTA cells were cultured similar to iSLK.RTA cells, with the addition of 1200 μg/mL hygromycin B (Thermo Fisher). Human umbilical vein endothelial cells (HUVECs; Lonza) were cultured in endothelial cell growth medium (EGM-2; Lonza). HUVECs were seeded onto gelatin (0.1% [wt/vol] in PBS)-coated tissue culture plates or glass coverslips.

### Lambda Red recombination for BAC16 mutagenesis.

All mutagenesis was carried out in the BAC16 background (58) supplied in the GS1783 strain of Escherichia coli (a generous gift from Jae Jung). All restriction enzymes were purchased from New England Biolabs (NEB) and all primers were purchased from Invitrogen.

A kanamycin resistance and levansucrase enzyme cassette (KanSacB) was amplified using primers (Table 1) with 40-bp overhanging sequences that correspond to the K12 sequence of the *kaposin* locus in the BAC16 genome. These primers also allowed for the introduction of BamHI and NotI restriction endonuclease sites. The resulting PCR product was then subcloned into pcDNA3.1+ (Addgene) using BamHI and NotI restriction endonucleases (pcDNA3.1+-KanSacB).

**TABLE 1:** BAC16 mutagenesis primers

Primer	Orientation^*a*^	Sequence (5′–3′)^*b*^
KanSacB homology	F	ATACAT**GGATCC***AGGATGACGACGATAAGTAGGGATAACAGGGTAAT*
	R	ATTAGT**GCGGCCGC**GGACATGAACTGCCACAAACACCGTTAAGCCTCTATCCAT*CAACCAATTAACCAATTCTGAT*
altKapC homology	F	ATACAT**GCTAGC**CGGAGGACGGATCTCTTGGATTTACACGTATCGAGGAGCG*GTAGCACCCCAGGAACCCGTCCTAGCACACCCCA*
	R	TGATTA**GGATCC**GGACATGAACTGCCACAAACACCGTTAAGCCTCTATCCATA*CACTGAGATTGAAGAG*
altKapB homology	F	ATACAT**GCTAGC**CGGAGGACGGATCTCTTGGATTTACACGTATCGAGGAGCG*GTGGCACCCCAGGAACCCGT*
	R	TGATTA**GGATCC**GGACATGAACTGCCACAAACACCGTTAAGCCTCTATCCAT*GCATAGGTATAGGTGGTAGA*
Kaposin screening	F	*CAGGAAACGCTATAAAGAAGAGG*
	R	*ACAGACAAACGAGTGGTGGTATC*
altKapC screening	R	5′-*ATTACCTGGAAGCAATGCACC*-3′
	R	*TGATGAAGGAACTAGATTGCCAG*
KanSacB screening	F	*GCGACAACCATACGCTGAGAG*
Revertant screening (homology primer)	R	*ACACCGTTAAGCCTCTATCCAT*
Revertant screening (revert primer)	R	*TGCATTGGGATTGGAGTGAGGA*

a F, forward; R, reverse.

b Restriction enzyme recognition sites are in boldface. BAC16 homology regions are underlined. Complementary target sequences are italicized.

To create single deletions of KapB and KapC, two direct repeat (DR) region gene blocks were synthesized (BioBasic) where the third nucleotide of each codon of DR1 and DR2 was altered to insert premature stop codons and nonsynonymous mutations into one frame, while creating synonymous mutations in the other. This allowed the primary amino acid sequence of either KapB or KapC to be maintained while eliminating expression from the other ORF and vice versa. Primers were used to amplify the gene block with 40-bp overhanging sequences that (i) flank the gene block, (ii) are homologous to the upstream and downstream regions of the DRs in the BAC16 genome, and (iii) introduce NheI and BamHI restriction sites. The resulting PCR product was then subcloned into pcDNA3.1+-KanSacB (described above) using NheI and BamHI restriction sites (pcDNA3.1+-geneblock-KanSacB).

Lambda Red recombination was conducted in the GS1783 *E. coli* strain, which expresses the I-Sce enzyme and, in a temperature (42°C)-dependent manner, the lambda Red recombinase genes (beta, exo, and gam). BAC16-GS1783 E. coli were cultured at 30°C in lysogeny broth (LB) containing 30 μg/mL chloramphenicol (Sigma-Aldrich) until reaching an optical density at 600 nm (OD_600_) of 0.6 to 0.8. Cultures were incubated for 30 min at 42°C prior to pelleting at room temperature at 3,000 × *g* for 5 min. Bacterial pellets were resuspended in ddH_2_O and pelleted again at 3,000 × *g* for 5 min. This step was repeated once more, following which bacterial pellets were resuspended in 50 μL of ddH_2_O and placed on ice. Meanwhile, pcDNA3.1+-geneblock-KanSacB was digested with NheI and NotI restriction endonucleases to generate a linear DNA fragment for recombination. This was purified by 1% agarose gel electrophoresis and gel extracted according to QIAquick gel extraction kit (Qiagen) standard protocol. The purified fragment was electroporated (2.5 kV, 25 μF Bio-Rad Gene Pulser II) into 50 μL of ddH_2_O-washed BAC16-GS1783 *E. coli*. Bacteria was recovered for 1 h in 1 mL of LB medium at 30°C, plated onto LB agar plates containing 50 μg/mL kanamycin, and incubated at 30°C overnight (O/N). Colonies were screened for successful recombination using colony PCR and two sets of screening primers designed to assess insert size and orientation (Table 1).

Colonies that produced bands of the expected size with both primer sets were inoculated into 1 mL of LB containing 1% arabinose (Sigma-Aldrich) for 1 h at 30°C to induce I-Sce expression to linearize the inserted KanSacB cassette. Bacteria were then incubated at 42°C for 30 min to induce recombinase gene expression and recovered for 1 h at 30°C prior to plating on LB agar plates containing 30 μg/mL chloramphenicol (Sigma-Aldrich) and 5% sucrose (Sigma-Aldrich), which selects for the loss of SacB that synthesizes a toxic compound in the presence of sucrose. Positive colonies were replica plated on LB agar plates containing 50 μg/mL kanamycin. Colonies that grew on both chloramphenicol- and sucrose-containing plates and which were no longer kanamycin resistant were screened by colony PCR for having undergone the second recombination to remove the KanSacB cassette, and colony PCR products were verified by Sanger sequencing using the primers in Table 1.

### Isolation of BACmid DNA.

BACmid DNA was isolated from GS1783 *E. coli* cultures of 5 mL of LB containing 30 μg/mL chloramphenicol grown O/N at 30°C. Bacteria was pelleted at 3,000 × *g* for 5 min, resuspended in buffer P1 containing RNase A, lysed using alkaline lysis in buffer P2, and neutralized with buffer N3 (All reagents from QIAprep kits; Qiagen). After the addition of buffer N3, lysates were placed on ice for 10 min prior to centrifugation at 17,000 × *g* for 10 min to pellet precipitate. A 750-μL portion of supernatant was then mixed with 750 μL of 100% isopropanol and placed on ice for 10 min prior to centrifugation at 17,000 × *g* for 10 min to pellet the DNA. Supernatants were removed, and the DNA pellet was left to air dry before being resuspended in 50 μL of 10 mM Tris-Cl (pH 8.5). DNA was used immediately for transfection.

### Whole-genome sequencing of KSHV BACs.

BACmid DNA was isolated from GS1783 *E. coli* cultures of 400 mL of LB containing 30 μg/mL chloramphenicol grown O/N at 30°C. Bacteria was pelleted at 4,000 × *g* for 10 min at 4°C. DNA was then extracted using the Qiagen large construct kit in accordance with manufacturer’s protocol. Critically, this isolation includes an exonuclease step which removes sheared *E. coli* DNA, thereby allowing greater coverage of the BAC16 genome.

Whole-genome sequencing was performed by the Centre for Health Genomics and Informatics (University of Calgary, Calgary, Alberta, Canada). Briefly, libraries were prepared using an NEB Ultra II DNA Library Prep kit for Illumina (NEB), quantified using a Kapa qPCR Library kit (Roche), and sequenced using a MiSeq 500 v2 nano run (MiSeq reagent kit v2; Illumina) to generate 250 + 250-bp paired end sequences. Raw reads were imported into Geneious Prime (61), paired and trimmed according to default settings. Reads were then aligned to the BAC16 reference genome (NCBI accession GQ994935). The average coverage was between ∼670× and 1,400×. Variations were identified based on a minimum coverage of 5 and a minimum frequency in variation of 0.25.

### Restriction length fragment polymorphism.

Isolated BACmid DNA was digested overnight using NheI restriction enzyme (New England Biolabs) at 37°C. Digested DNA fragments were then subjected to 1% agarose pulsed-field gel electrophoresis (PFGE; Bio-Rad) using 0.5% Tris/borate/EDTA (TBE) buffer and the following conditions: 6 V/cm, 120° field angle, and a switch time linearly ramped from 1 to 5 s over 14 to 15 h (CHEF DR III; Bio-Rad). DNA bands were visualized by staining the gel with a solution of 0.5 μg/mL ethidium bromide; excess stain was washed away with deionized water, and the gel was imaged on a ChemiDoc Touch imaging system (Bio-Rad). A DNA molecular weight standard (Bio-Rad; CHEF DNA, size, 8 to 48 kb) was used as reference.

### Generation of stable BAC16-iSLK.RTA cell lines.

Six-well plates of 70% confluent 293T cells in antibiotic-free 10% FBS–DMEM were washed once with phosphate-buffered saline (PBS), and medium was replaced with 1 mL of serum-free, antibiotic-free DMEM per well prior to transfection. Then, 5 μl of resuspended BAC16 DNA was incubated with 100 μl of Opti-MEM reduced serum medium (Thermo Fisher) for 5 min. Separately, 18 μl of polyethylenimine (PEI; Polysciences) was incubated with 100 μl of Opti-MEM, also for 5 min. These two mixtures were combined, incubated for 15 min, and added dropwise to each well. Cells were incubated at 37°C for 4 h, at which time the medium was replaced with antibiotic-free 10% FBS–DMEM before further incubation at 37C. This transfection procedure was used to create latent 293T stable cell lines shown in Fig. 3.

At 48 h posttransfection, cells were expanded into 10-cm dishes. One day later, the medium was removed and replaced with fresh antibiotic-free 10% FBS–DMEM containing 20 μg/mL hygromycin (Thermo Fisher Scientific). The concentration of hygromycin was steadily increased until a final concentration of 100 μg/mL was reached. When a confluent monolayer of hygromycin-resistant BAC16 293T cells was achieved, the cells were split once prior to liquid N_2_ storage in 90% FBS and 10% dimethyl sulfoxide (DMSO; Sigma-Aldrich).

To generate latent iSLK cells, each population of BAC16-transfected 293T cells was individually cocultured with puromycin-resistant iSLK.RTA cells in a 6-well dish at a 2:1 ratio. Once cells reached 80% confluence, the medium was replaced with antibiotic-free 10% FBS–DMEM containing 1 mM sodium butyrate (NaB; Sigma-Aldrich) and 20 ng/mL 12-*O*-tetradecanoylphorbol-13-acetate (TPA; Sigma-Aldrich) to induce latent BAC16-293T cells to produce progeny virions. At 96 h postreactivation, cells were expanded into 10-cm dishes. One day following expansion, the medium was replaced with antibiotic-free 10% FBS–DMEM containing 10 μg/mL puromycin and 100 μg/mL hygromycin. The hygromycin concentration was steadily increased in approximately 50-μg/mL increments until a final concentration of 240 μg/mL was reached. Once a near-confluent monolayer of BAC16-iSLK.RTA cells was achieved, the cells were split once prior to liquid N_2_ storage, as described above. This procedure was used to create the latent iSLK cell lines shown in Fig. 3. A single latent iSLK cell line was created for ΔKapABC, ΔKapBC, and ΔKapC, while we derived four latent iSLK cell lines for ΔKapB independently, as shown in Fig. 5.

### Production of KSHV BAC16 wild-type and recombinant virus.

Each population of BAC16-iSLK cells were reactivated using 1 μg/mL doxycycline (Dox; Sigma-Aldrich) and 1 mM NaB in antibiotic-free 10% FBS–DMEM. At 72 or 96 h postinfection, supernatants were collected and clarified at 4,000 × *g* to remove cellular debris and virus-containing supernatant stored at −80°C prior to use.

### *De novo* infection of naive HEK293T of HUVEC cells.

Subconfluent (50 to 70%) 12-well plates of 293T or HUVEC cells were incubated with viral inoculum diluted in antibiotic and serum-free DMEM containing 8 μg/mL hexadimethrine bromide (Polybrene; Sigma-Aldrich). Plates were centrifuged at 800 × *g* for 2 h at room temperature (108), the inoculum was removed and replaced with either antibiotic-free 10% FBS–DMEM (293T) or full EGM-2 (HUVECs). Cells were then returned to the 37°C incubator.

### Immunofluorescence.

Cells were seeded onto 18-mm-round coverslips (catalog no. 1.5; Electron Microscopy Sciences), infected or treated as noted for each experiment and fixed for 10 min at room temperature in 4% (vol/vol) paraformaldehyde (Electron Microscopy Sciences) in PBS. Samples were permeabilized with 0.1% (vol/vol) Triton X-100 (Sigma-Aldrich) in PBS for 10 min at room temperature and blocked in 1% human AB serum (Sigma-Aldrich) for 1 h at room temperature. Primary antibody was diluted in 1% human AB serum (Sigma-Aldrich) in PBS at the concentrations in Table 2 and incubated overnight. The following day secondary antibody was likewise diluted in 1% human AB serum, alongside Hoechst (Thermo Fisher Scientific) for nuclear staining, at the concentrations in Table 2 and incubated for 1 h. Samples were mounted with Prolong Gold AntiFade mounting media (Thermo Fisher Scientific). Images were captured using a Zeiss AxioObserver Z1 microscope with the 40× objective unless otherwise stated.

**TABLE 2:** Antibodies

Antibody	Species	Source or vendor, catalog no.	Application	Dilution
Hedls	Mouse	Santa-Cruz, sc-8418	Immunofluorescence	1:1,000
KapB	Rabbit	A generous gift from Don Ganem	Immunoblot	1:1,000
LANA	Rabbit	A generous gift from Don Ganem	Immunofluorescence	1:1,000
			Immunoblot	1:1,000
Alexa Fluor 647 phalloidin	NA^*a*^	Thermo Fisher, A22287 (1)	Immunofluorescence	1:100
Alexa Fluor 488	Chicken anti-rabbit	Thermo Fisher, A-21441	Immunofluorescence	1:1,000
Alexa Fluor 647	Chicken anti-mouse	Thermo Fisher, A-21463	Immunofluorescence	1:1,000
Hoechst 33342	NA	Thermo Fisher, H3570	Immunofluorescence	1:10,000
Beta-actin HRP conjugate	Rabbit	Cell Signaling Technologies, 5125	Immunoblot	1:8,000
Anti-rabbit IgG HRP linked	NA	Cell Signaling Technologies, 7074	Immunoblot	1:3,000
Anti-DNA-RNA hybrid antibody clone S9.6	Mouse	Millipore MABE, 1095	Drip	1:10

a NA, not applicable.

### Image analysis.

Image analysis was performed using CellProfiler (cellprofiler.org), an open-source software for image analysis (109). Quantification of Hedls/EDC4 and LANA-nuclear body (NB) puncta was performed as previously described (110) with the following modifications. Rather than staining cells with wheat germ agglutinin to identify cell periphery, individual cell cytoplasms were defined based on a 150-pixel propagation away from the Hoechst-stained nucleus. The number of Hedls/EDC4 puncta within a defined cell cytoplasm was reported. To quantify the number of puncta in KSHV-infected cells, a gating strategy was applied to include only cells with LANA-positive nuclei and defined cell cytoplasms for Hedls/EDC4 puncta quantification. LANA NBs were defined by thresholding and size was measured using the “MeasureObjectSizeShape” function, respectively. Object area refers to the number of pixels within the identified region. At least 50 cells were quantified for each condition.

### Immunoblotting.

Cells were lysed in 1× Laemmli buffer (4% SDS, 20% glycerol, 120 mM Tris-Cl [pH 6.8] and ddH_2_O) and stored at −20°C until use. The DC protein assay (Bio-Rad) was used to quantify total protein concentration as per the manufacturer’s instructions.10 to 15 μg of protein lysate was resolved by SDS-PAGE on TGX Stain-Free acrylamide gels (Bio-Rad). Membranes were blocked in 5% bovine serum albumin (BSA) or 5% skim milk in Tris-buffered saline-Tween 20 (TBS-T). Primary and secondary antibodies were diluted in 2.5% BSA or 2.5% skim milk, dilutions can be found in Table 1. Membranes were visualized using ProtoGlow ECL (National Diagnostics) and the ChemiDoc Touch Imaging system (Bio-Rad).

### Intracellular viral genome qPCR.

Confluent 6-well plates of BAC16-iSLK.RTA cells were lysed using a DNeasy blood and tissue kit (Qiagen) according to the manufacturer’s protocol. Isolated DNA was stored at −20°C until further use. DNA was diluted 1:30 prior to qPCR amplification using SsoFast EvaGreen Supermix (Bio-Rad) and viral ORF26 or cellular β-actin (control) primers as listed in Table 3. Fold change in viral genome copy number was determined using the “ΔΔ-Quantitation Cycle” (Cq) method.

**TABLE 3:** qPCR primers

Primer	Orientation^*a*^	Sequence (5′–3′)
ORF26	F	CAGTTGAGCGTCCCAGATGA
	R	GGAATACCAACAGGAGGCCG
β-actin	F	CTTCCAGCAGATGTGGATCA
	R	AAAGCCATGCCAATCTCATC
Luc	F	TGCGCAAGAATAGCTCCTCC
	R	TTCGGCAACCAGATCATCCC
IL-6	F	GTCCAGTTGCCTTCTCCCTGG
	R	GAGATGCCGTCGAGGATGTACC
CSF2	F	AAATGTTTGACCTCCAGGAGCC
	R	ATCTGGGTTGCACAGGAAGTT
VEGFA	F	CGAGACCTTGGTGGACATC
	R	CTGCATGGTGACGTTGAAC
IL-1β	F	CTCTCACCTCTCCTACTCACTT
	R	TCAGAATGTGGGAGCGAATG
LANA	F	GCGAAGGAAGCATGTCGCC
	R	CACAGGATGGGATGGAGGGATTG
18S	F	CGTTCTTAGTTGGTGGAGCG
	R	CCGGACATCTAAGGGCATCA
HPRT-1	F	TGGCGTCGTGATTAGTGATG
	R	GACGTTCAGTCCTGTCCATAAT

a F, forward; R, reverse.

### DNase-protected viral genome qPCR.

Supernatants from reactivated iSLK cells were harvested and clarified at 4,000 × *g* for 5 min. Aliquots of 180 μL were stored separately at −80°C prior to use. Supernatants were thawed at 37°C and mixed with 20 μL of a 500 U of DNase I (NEB), 10× buffer, and nuclease-free water mixture, followed by incubation at 37°C for 30 min. DNA was then extracted by using a DNeasy blood and tissue kit with the following adjustments: per sample, 500 μg of salmon sperm DNA (Invitrogen) and 1 ng of a luciferase (*luc2*) containing plasmid (pGL4.26, Clontech) was added to buffer AL prior to lysis. DNA was not diluted prior to qPCR amplification using SsoFast EvaGreen Supermix (Bio-Rad) and viral ORF26 and *luc2* (control) primers as listed in Table 3. Since every viral particle contains one genome, and one genome contains a single copy of the ORF26 gene, the number of viral DNA molecules could be calculated. Fold change in viral DNase-protected genomes was determined using the Cq method.

### Lentivirus production and transduction.

All lentiviruses were generated using a second-generation system. Briefly, HEK293T cells were transfected with pSPAX2, MD2G, and the plasmid containing a gene of interest using PEI (Polysciences). psPAX2 was a gift from Didier Trono (Addgene, plasmid 12260), and pMD2.G was a gift from Didier Trono (Addgene, plasmid 12259). Viral supernatants were harvested 48 h posttransfection, clarified using a 0.45-μm polyethersulfone filter (VWR), and frozen at −80°C until use. For transduction, lentiviruses were thawed at 37°C and added to target cells in complete medium containing 5 μg/mL Polybrene (Sigma) for 24 h. The medium was changed to selection media containing 5 μg/mL blasticidin (Thermo), and cells were selected for at least 48 h before proceeding with experiments.

### Transfection.

Twelve-well plates of 70% confluent 293T cells in antibiotic-free 10% FBS–DMEM were washed once with PBS, and medium was replaced with 1 mL of serum-free, antibiotic-free DMEM per well prior to transfection. Cells were transfected with 1 μg of plasmids containing a gene of interest (Table 4) using PEI (Polysciences). At 4 h posttransfection, the medium was replaced with antibiotic-free 10% FBS–DMEM.

**TABLE 4:** Plasmids

Plasmid	Use	Source and/or reference	Mammalian selection
pcDNA3.1(+)	Empty vector control	Invitrogen, V79020 (79)	NA^*a*^
pcDNA3.1 KapB KS lung	Overexpression	C. McCormick (Dalhousie University)	NA
pcDNA3.1 KapB BAC16	Overexpression	Cloned from pUC57 KapB BAC16 (Bio Basic)	NA
pLJM1	Empty vector control	112	Blasticidin
pLJM1 KapB (pulmonary KS)	Overexpression	Cloned from pBMNIP-KapB (51) into pLJM1-BSD	Blasticidin
pMD2.G	Lentivirus generation	Addgene, 12259	NA
psPAX2	Lentivirus generation	Addgene, 12260	NA

a NA, not applicable.

### RT-qPCR.

RNA was collected using a RNeasy Plus minikit (Qiagen) according to the manufacturer’s instructions and stored at −80°C until further use. The RNA concentration was determined using a NanoDrop One^C^ (Thermo), and 500 ng total was reverse transcribed using qScript XLT cDNA SuperMix (QuantaBio) using a combination of random and oligo(dT) primers according to the manufacturer’s instructions. cDNA was diluted 1:10 for all RT-qPCR experiments and SsoFast EvaGreen Mastermix (Bio-Rad) was used to amplify cDNA. The Cq method was used to determine the fold change in expression of target transcripts. qPCR primer sequences can be found in Table 3.

### Fluorescent *in situ* hybridization for KSHV genomic DNA.

To visualize KSHV DNA in individual cells, Alexa Fluor 488-conjugated peptide nucleic acid (PNA) probes were designed and custom ordered from PNA Bio, Inc. The PNA probes were designed to hybridize against the TR sequence and contained the following sequences: probe 1, 5′-CCCTCTCTCTACTGTGCG-3′; and probe 2, 5′-GCCCTGGACACTACGTGA-3′. For the fluorescence *in situ* hybridization (FISH), the staining was performed as previously described (110). Briefly, iSLK cells were seeded on 12 mm glass coverslips for 24 h prior to fixation with 4% (vol/vol) paraformaldehyde containing 2% (wt/vol) sucrose for 10 min at room temperature. Coverslips were washed with 1× PBS, and the cells were permeabilized with 0.3% Triton X-100/1× PBS for 5 min at room temperature. Next, cells were blocked for 10 min with 2% (wt/vol) BSA and blocked for 30 min with 10% normal goat serum. After a washing step with 1× PBS, the coverslips were dehydrated using a series of ethanol concentrations (70, 80, 90, and 100%) and air dried before incubation with a hybridization solution containing 100 nM PNA probes 1 and 2. The coverslips were placed on the slide with the PNA probes, cells facing down, and denatured at 83°C for 8 min before incubation overnight in a humid, dark chamber at room temperature. Samples were washed twice for 15 min at room temperature with 70% (vol/vol) formamide/10 mM Tris-HCl (pH 7.2) with vigorous shaking and three times for 5 min at room temperature with 0.1 M Tris-HCl (pH 7.2)/0.15 M NaCl containing 0.05% (vol/vol) Tween 20 with shaking. DNA was counterstained with (4′,6′-diamidino-2-phenylindole; 1:10,000) in PBS for 8 min at room temperature, and the slips were cured overnight with Fluoromount-G mounting medium (Electron Microscopy Sciences). Z-stack images were acquired on a Nikon Eclipse Ti inverted epifluorescence microscope, using a 60×/1.4 N.A. oil objective and 0.2-μm step size. Images were deconvolved with Huygens Essential version 18.10 (Scientific Volume Imaging, The Netherlands), using the classic maximum-likelihood estimation (CMLE) algorithm, with SNR: 40 and 50 iterations. Deconvolved images were analyzed using FIJI/ImageJ (10.1038/nmeth.2019), and the total PNA FISH fluorescence signal within the nucleus was thresholded, quantified, and plotted.

### DNA-RNA immunoprecipitation.

For DNA-RNA immunoprecipitation (DRIP), the procedure was performed as previously described (111). In brief, iSLK cells were trypsinized and harvested. Cell pellets were resuspended and lysed overnight at 37°C in 20 mM Tris (pH 8), 4 mM EDTA, 20 mM NaCl, 0.3 μg/μL proteinase K (Invitrogen), and 1% SDS. Genomic DNA was extracted by phenol-chloroform/isoamyl alcohol (25:24:1; Invitrogen) extraction using MaXtract high-density phase-lock tubes (Qiagen) and precipitated using 100% (vol/vol) ethanol and 3 M sodium acetate (pH 5.2). After being washed with 70% ethanol and air drying, the DNA was gently resuspended in TE and left on ice for a couple hours to allow all the DNA to resolve. Next, the genomic DNA was digested overnight at 37°C using a restriction enzyme cocktail (30 U of SspI, BsrgI, XbaI, EcoRI, and HindIII (all NEB), 1× BSA (VWR) and 1 mM Spermidine [Invitrogen]). After confirming successful digestion on a 1% agarose gel, the DNA was ethanol precipitated using light phase-lock tubes (VWR), and 8 μg of DNA was treated with or without RNase H (2 U/1 μg of DNA; NEB) in 1× RNase H buffer (NEB) at 37°C for 4 h. After the input sample was collected, each DNA sample was diluted in 1× binding buffer (10 mM sodium phosphate [pH 7], 0.14 M NaCl, and 0.05% [vol/vol] Triton X-100), followed by incubation with 5 μg of S9.6 antibody overnight at 4°C while rotating for the DNA:RNA immunoprecipitation. Next, magnetic protein A/G beads (Bio-Rad) were washed with 1× binding buffer and added to the DNA/antibody complexes for 2 h at 4°C while rotating. Bound beads were washed with 1× binding buffer, and elution was performed in 50 mM Tris (pH 8), 10 mM EDTA, 0.5% (vol/vol) SDS, and 0.3 μg/μL proteinase K for 1 h at 55°C with regular shaking. After separating the magnetic beads from the lysates, the DNA was purified as described previously and resolved in Tris-EDTA. To determine the amount of RNA:DNA hybrids at each locus, qPCR was performed using SYBR green PowerUp master mix (Thermo) and locus-specific primers (Table 5) on an Applied Biosystem QuantStudio 6 Flex machine.

**TABLE 5:** DRIP qPCR primers

Primer	Orientation^*a*^	Sequence (5′–3′)
RPL13A	F	AGGTGCCTTGCTCACAGAGT
	R	GGTTGCATTGCCCTCATTAC
EGR1	F	GAACGTTCAGCCTCGTTCTC
	R	GGAAGGTGGAAGGAAACACA
ORF45	F	GGGAGGTGACCCTTTGTGCT
	R	CTCATGGACGTGGGCCAGA
T1.4	F	CAAGCACACAACCCCTGCTA
	R	GGGTTATATGCGCGTGCTTG
Kaposin 5′	F	GGGGGTACTACCTGGTTCCT
	R	CACGGAGGACGGATCTCTTG
Kaposin 3′	F	GGATGTGTTACTAAATGGGTGGCG
	R	GCAACTCGTGTCCTGAATGCTAC

a F, forward; R, reverse.

### Statistics.

Data shown are the means ± standard deviations (SD). Unless indicated otherwise in the figure legends, the statistical significance was determined using a repeated measures one-way analysis of variance (ANOVA) using Dunnett’s *post hoc* test. *P* values are indicated in figures (*, *P < *0.05; **, *P < *0.01; ***, *P < *0.001; ****, *P < *0.0001; ns, nonsignificant). All statistics were performed using GraphPad Prism version 9.0.1.

### Data availability.

All BAC16 virus sequences were deposited in the NCBI Sequence Read Archive (SRA) and are accessible through the following SRA accession numbers: SAMN19671530 (WT KSHV BAC16), SAMN19671876 (KSHV BAC16 ΔKapABC), SAMN19671877 (KSHV BAC16 ΔKapBC), SAMN19671902 (KSHV BAC16 ΔKapB), and SAMN19672118 (KSHV BAC16 ΔKapC).
